# Epigenetic regulation of cell state by H2AFY governs immunogenicity in high-risk neuroblastoma

**DOI:** 10.1172/JCI175310

**Published:** 2024-09-10

**Authors:** Divya Nagarajan, Rebeca T. Parracho, David Corujo, Minglu Xie, Ginte Kutkaite, Thale K. Olsen, Marta Rubies Bedos, Maede Salehi, Ninib Baryawno, Michael P. Menden, Xingqi Chen, Marcus Buschbeck, Yumeng Mao

**Affiliations:** 1 Science for Life Laboratory, Department of Immunology, Genetics and Pathology and; 2 Department of Immunology, Genetics and Pathology, Uppsala University, Uppsala, Sweden.; 3 Program of Myeloid Neoplasms, Program of Applied Epigenetics, Josep Carreras Leukaemia Research Institute (IJC), Campus Can Ruti Site, Badalona, Spain.; 4 Computational Health Center, Helmholtz Munich, Neuherberg, Germany.; 5 Department of Biology, Ludwig-Maximilians University Munich, Martinsried, Germany.; 6 Childhood Cancer Research Unit, Department of Women’s and Children’s Health, Karolinska Institutet, Solna, Sweden.; 7 Department of Biochemistry and Pharmacology, University of Melbourne, Melbourne, Australia.

**Keywords:** Immunology, Oncology, Cancer, Cancer immunotherapy

## Abstract

Childhood neuroblastoma with *MYCN* amplification is classified as high risk and often relapses after intensive treatments. Immune checkpoint blockade therapy against the PD-1/L1 axis shows limited efficacy in patients with neuroblastoma, and the cancer intrinsic immune regulatory network is poorly understood. Here, we leverage genome-wide CRISPR/Cas9 screens and identify *H2AFY* as a resistance gene to the clinically approved PD-1 blocking antibody nivolumab. Analysis of single-cell RNA-Seq datasets reveals that *H2AFY* mRNA is enriched in adrenergic cancer cells and is associated with worse patient survival. Genetic deletion of *H2afy* in *MYCN*-driven neuroblastoma cells reverts in vivo resistance to PD-1 blockade by eliciting activation of the adaptive and innate immunity. Mapping of the epigenetic and translational landscape demonstrates that *H2afy* deletion promotes cell transition to a mesenchymal-like state. With a multiomics approach, we uncovered *H2AFY*-associated genes that are functionally relevant and prognostic in patients. Altogether, our study elucidates the role of *H2AFY* as an epigenetic gatekeeper for cell states and immunogenicity in high-risk neuroblastoma.

## Abstract 2

H2AFY controls immunogenicity in neuroblastoma.

## Graphical abstract

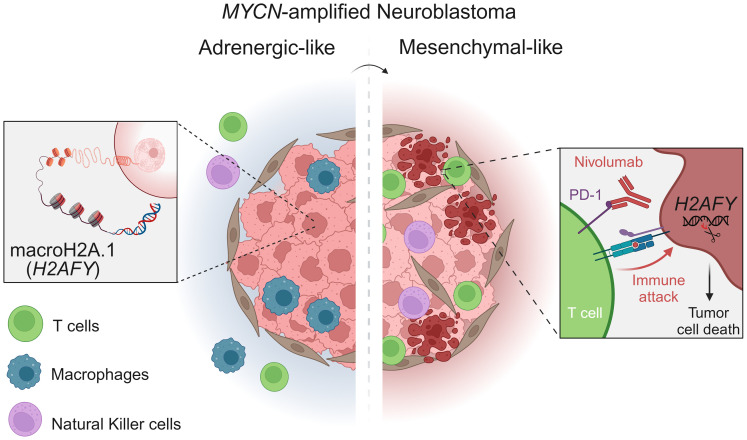

## Introduction

High-risk neuroblastoma (NB) with amplification of the *MYCN* oncogene is an aggressive extra-cranial solid tumor in infants and young children, which accounts for 20% of the total disease cases (1). Despite recent advances in multimodal therapies, the prognosis for NB continues to be poor, with less than 50% of patients remaining relapse-free 5 years after diagnosis (2). Moreover, the current intensive treatment options pose long-term challenges to the quality-of-life in survivors of NB (3). Therefore, new treatment options with selective eradication of cancer cells are urgently needed to improve the quality of life in children with high-risk NB.

Immune checkpoint blockade (ICB) therapy against negative regulators of the immune system, e.g., CTLA-4, PD-1, or PD-L1, has shown clinical benefits leading to their approval as anticancer drugs in several adult cancer types (4). ICB therapy has also been tested in children with NB but only limited clinical activity was observed (5, 6).

Mutational burden in cancer cells is recognized as a key factor for antitumor immunity and has been tested as a predictive biomarker for immunotherapy (7, 8). Moreover, multiple clinical trials demonstrate that patients with PD-L1–positive tumors are more likely to benefit from ICB drugs against the PD-1/L1 axis (7, 9).

NB tumors exhibit a lower mutational burden than adult cancer types that are responsive to ICB therapy (10, 11). In a large cohort analysis consisting of samples from 254 patients with NB, only 3% of samples show high membrane PD-L1 protein expression by IHC staining, and PD-L1 positivity is more frequent in non-*MYCN* amplified tumors (12, 13). Moreover, *MYCN* amplification has been demonstrated to repress the expression of HLA class I molecules (14) and is associated with poor infiltration of immune cells (13, 15). In recent years, we and others have employed genome-wide CRISPR screens to uncover cancer intrinsic mechanisms controlling cytotoxicity mediated by human immune cells (16–18), but the intrinsic immune resistance program in high-risk NB cells is yet to be revealed.

Here, we have leveraged genome-wide CRISPR screens to identify resistance genes against ICB therapy in *MYCN*-amplified NB cells using a human coculture system. We believe that our work demonstrates that epigenetic reprogramming of NB cell states promotes cancer immunogenicity and reverted in vivo resistance to ICB therapy

## Results

### Human NB cells respond to IFNG stimulation.

**Figure 1:**
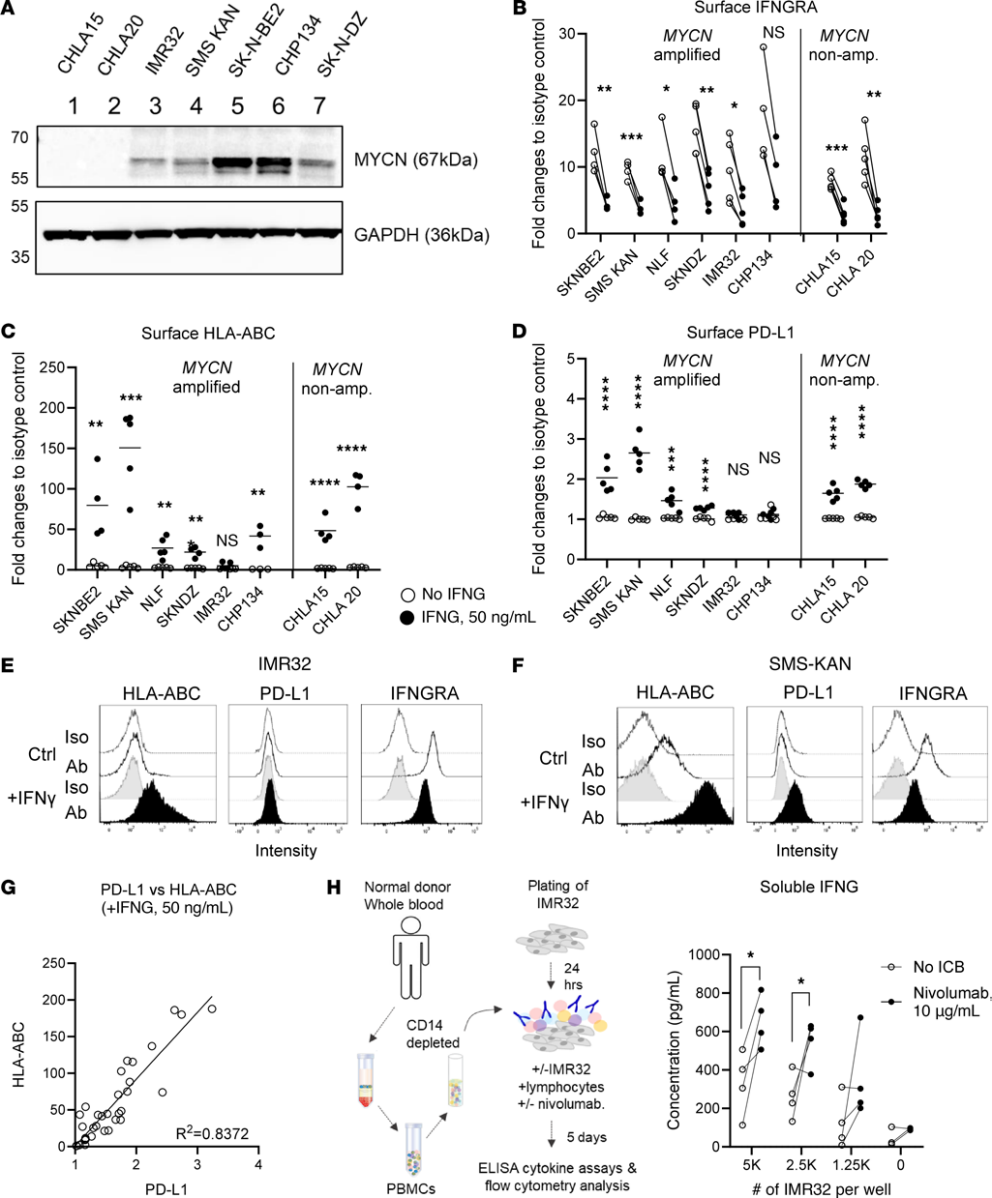
Human NB cells responded to IFNG stimulation. (**A**) Expression of the MYCN protein in a panel of human NB cell lines. Representative Western blot image of 3 biological repeats. Surface expression of (**B**) IFNGRA, (**C**) HLA-ABC, and (**D**) PD-L1 on human NB cell lines +/– 50 ng/mL recombinant human IFNG (rhIFNG) after 16–18 hours treatment. Each dot represents an independent experiment of at least 4 biological repeats. Representative histograms of HLA-ABC, PD-L1, and IFNGRA in (**E**) IMR32 and (**F**) SMS-KAN cells. (**G**) Correlation between surface HLA-ABC and PD-L1 on NB cell lines treated with rhIFNG. (**H**) Monocytes were depleted from PBMCs isolated from healthy blood donors and the unsorted lymphocytes were cocultured with IMR32 cells, with or without 10 μg/mL nivolumab. After 5 days, the levels of soluble IFNG released into culture supernatants were determined by ELISA. Primary lymphocytes cultured alone were used as controls. Each dot represents results from an independent donor (*n* = 4), unpaired 2-tailed *t* test. *P < 0.05; **P < 0.01; ***P < 0.001; ****P < 0.0001.

To investigate the immunological features of human NB cells, we utilized a panel of human NB cell lines with *MYCN* amplification, i.e. IMR32, SMS-KAN, SK-N-BE2, CHP134, and SK-N-DZ, and 2 non-*MYCN* amplified cell lines as controls, i.e. CHLA-15 and -20 (Figure 1A). While PD-L1 was absent on the surface of all NB cell lines, 5 out of 6 *MYCN*-amplified NB cell lines showed baseline expression of HLA-ABC that was comparable to the non-*MYCN* amplified cell lines (Supplemental Figure 1A; supplemental material available online with this article; https://doi.org/10.1172/JCI175310DS1).

Because activation of the IFNG/JAK/STAT pathway is linked to immunotherapy response (19), we evaluated the responsiveness of human NB cells to recombinant human IFNG in vitro. All NB cell lines demonstrated high baseline surface expression of functional IFNGRA, which was significantly downregulated upon cytokine binding (Figure 1B). Moreover, the majority of NB cell lines upregulated surface HLA-ABC and PD-L1 expression in response to IFNG treatment (Figure 1, C, D, and F). The strongest induction of surface HLA-ABC and PD-L1 was observed in SK-N-BE2 and SMS-KAN cell lines, while NLF, SK-N-DZ, and CHP134 demonstrated weak responses (Figure 1, C and D). In contrast, IMR32 cells failed to respond to IFNG stimulation (Figure 1, C–E). Among all human NB cell lines tested, we observed a strong correlation between HLA-ABC and PD-L1 protein expression upon IFNG treatment (Figure 1G), indicating that *MYCN*-amplified human NB cells harbor an intact IFNG-signaling pathway.

Because IMR32 cells demonstrated an immune-resistant phenotype, we tested cancer-driven immune activation in a human tumor-immune coculture system (TICS) that was designed to model the response and resistance to clinically approved drugs against the PD-1/L1 axis (18, 20). As shown in Figure 1H, the induction of IFNG from human lymphocytes by IMR32 cells was dependent on the effector-to-target (E:T) ratios between lymphocytes and IMR32 cells. The addition of a clinically approved PD-1 blocking drug, nivolumab, enhanced the release of IFNG (Figure 1H) and granzyme B (Supplemental Figure 1B) in TICS. Our data suggested that *MYCN*-amplified human NB cells were capable of responding to stimulatory cytokines and PD-1 blockade.

### Genome-wide CRISPR/Cas9 screens identify NB genes to sensitize nivolumab.

**Figure 2:**
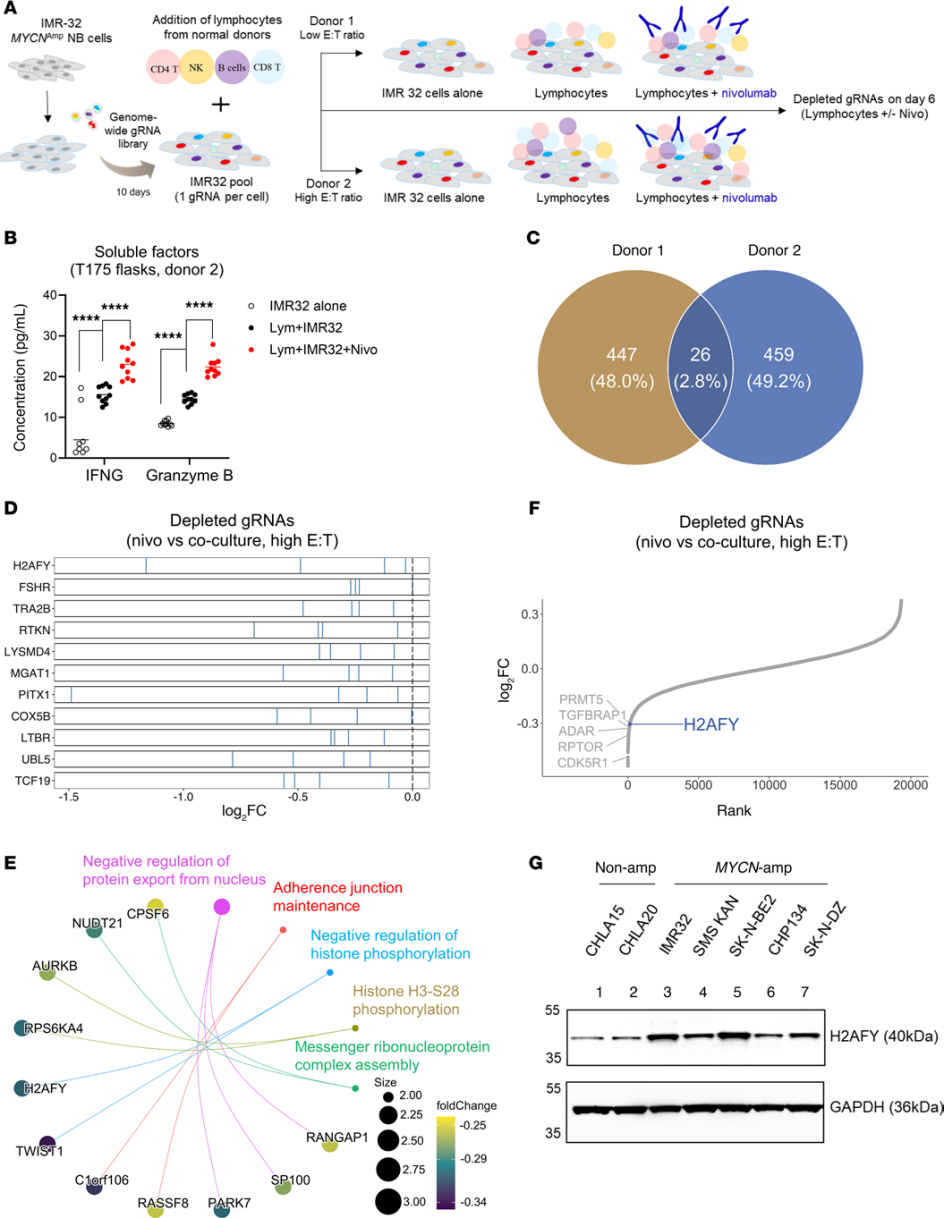
Genome-wide CRISPR/Cas9 screens identified *H2AFY* as a resistance gene to nivolumab in human NB cells. (**A**) Schematic illustration of the setup of CRISPR screens using an IMR32/lymphocyte coculture. (**B**) Culture supernatants were collected from the flasks at the end of the CRISPR screen using donor 2. Levels of soluble IFNG and granzyme B were quantified using ELISA. (**C**) Venn diagram to illustrate the top and commonly depleted genes from the screens when comparing cocultures treated with or without nivolumab. (**D**) Performance of the 4 individual gRNAs against the top commonly depleted genes. (**E**) Functional enrichment analysis to capture pathways represented by the top depleted genes in the CRISPR screens. (**F**) Ranking of known immune resistance genes and *H2AFY* in the genome-wide CRISPR screen performed with the high E:T ratio, when comparing nivolumab treated and nontreated cocultures. (**G**) Detection of the H2AFY protein was performed simultaneously as the detection of MYCN using Western blotting in a panel of human NB cell lines. The GAPDH bands were identical to the ones in Figure 1A. Representative blot from 2 biological repeats, unpaired 2-tailed *t* test. ****P < 0.0001.

Next, we sought to reveal NB cancer intrinsic genes that controlled nivolumab response in genome-wide CRISPR screens using our established workflow (Figure 2A) (18). In brief, Cas9-expressing IMR32 cells transduced with the Brunello gRNA library were cocultured with freshly isolated human lymphocytes with or without nivolumab for 6 days, followed by the determination of gRNA frequencies in surviving cancer cells. We focused on the comparison between cocultures with or without nivolumab in order to uncover resistance genes specific to ICB therapy. Two independent screens were performed at high or low E:T ratios in order to identify common resistance genes. Because IMR32 cells were poorly immunogenic, immune-mediated cancer cell death in TICS was not sufficiently high to allow accurate assessment of enriched genes.

To confirm that activation of lymphocytes was enhanced by nivolumab in the genome-wide CRISPR/Cas9 screens, we sampled supernatants from all culture flasks and quantified levels of IFNG and granzyme B, respectively. In accordance with previous results, these soluble factors were released at significantly higher levels in the coculture than in IMR32 cells alone and were further enhanced when nivolumab was present (Figure 2B).

Using a cut-off of 2 SD below the mean of gene essentiality score distribution, we observed 26 commonly depleted genes between 2 screens (Figure 2C and Supplemental Data 1) with 473 and 485 depleted genes from donor 1 and donor 2, respectively (Supplemental Figure 1, C and D). Next, we examined the performance of the 4 individual gRNAs against the same gene in the screen with a high E:T ratio and shortlisted genes where all 4 gRNAs consistently showed a negative log_2_ fold change, resulting in 11 genes: *H2AFY, FSHR, TRA2B, RTKN, LYSMD4, MGAT1, PITX1, COX5B, LTBR, UBL5,* and *TCF19* (Figure 2D). Functional pathway analysis revealed that genes governing epigenetic regulation of histone phosphorylation were enriched among the depleted genes (Figure 2E). Additionally, we identified known genes or pathways that control immunogenicity in cancer cells, including *PRMT5* (18, 21), *ADAR* (22), cell cycle (*CDK5R1, RPTOR*), and the immune suppression related gene *TGFBRAP1* (Figure 2F).

When tested for protein expression, H2AFY was detected in a panel of human NB cell lines (Figure 2G) and we observed stronger H2AFY protein expression in *MYCN*-amplified NB cell lines (IMR32, SMS-KAN, SK-N-BE2, CHP134, and SK-N-DZ), compared with the nonamplified CHLA-15 and CHLA-20 lines (Figure 2G). Because gRNAs against *H2AFY* showed a robust performance and the protein was expressed in NB cell lines, we selected this gene as the target for biological validation.

### H2AFY sustains the adrenergic cell status of NB cells.

**Figure 3:**
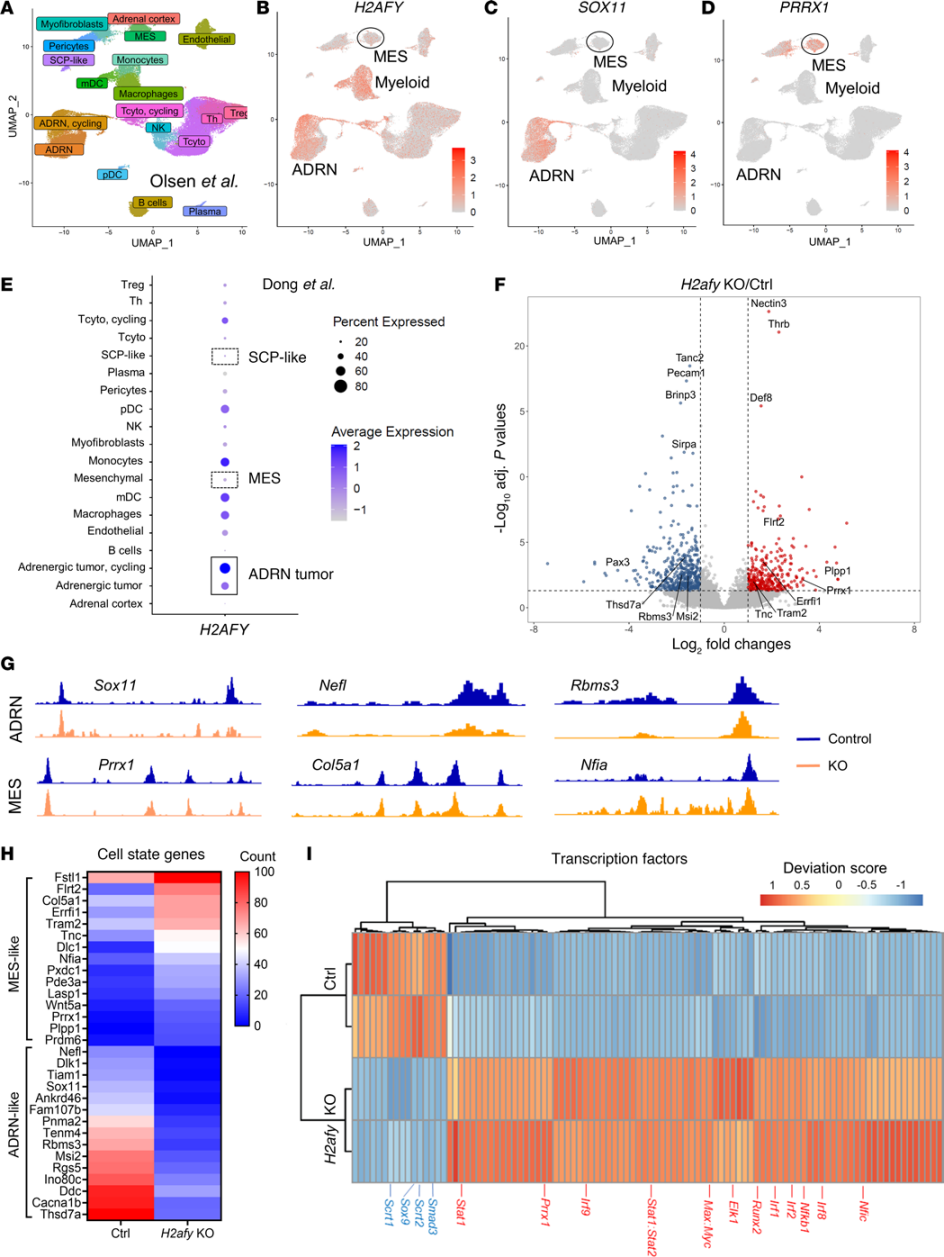
*H2AFY* sustained the ADRN cell state in *MYCN*-driven NB. (**A**) scRNA-Seq was performed in human NB tumors and cell subsets were annotated (25). Expression of (**B**) *H2AFY*, (**C**) *SOX11* and (**D**) *PRRX1* was visualized. (**E**) Expression of the *H2AFY* mRNA in different cell subsets was visualized in an independent scRNA-Seq dataset (26) using the same annotation. The chromatin accessibility of genes in control or *H2afy* CRISPR-KO 9464D cells was mapped using ATAC-Seq. (**F**) Volcano plot for peaks showing the most significant epigenetic accessibility. (**G**) Epigenetic profile for representative genes for the ADRN and MES cell state. (**H**) Heatmap for genes associated with cell state using ATAC-Seq read counts. (**I**) Chromatin accessibility for transcription factors (TF) in control or KO cells. Peaks were selected by using the DESeq2 package with *P*_adj_ < 0.05 and absolute log_2_ FC > 1. TF motif enrichment was performed with ChromVar based on the selected peaks and visualized using the deviation scores. The top 100 peaks were visualized in heatmap based on the variability.

H2AFY is a core histone variant that can replace the replication-coupled H2A histone in the nucleosome in a locus-specific fashion (23). In contrast to other histones, the H2AFY protein has a unique tripartite structure consisting of a histone fold, an unstructured linker domain, and a globular macrodomain (24). To explore the clinical relevance of *H2AFY* mRNA in patients with NB, we analyzed 2 single-cell RNA-Seq (scRNA-Seq) datasets of NB tumors. Our analysis (25) depicted the intratumoral heterogeneity of human NB (Figure 3A). *H2AFY* mRNA was found in adrenergic (ADRN) cancer cells but was absent in cells at the mesenchymal-like (MES-like) or the schwann cell precursor–like (SCP-like) states (Figure 3B).

When comparing to known signature genes linked to the MES and ADRN cell states, we observed that *H2AFY*-positive NB cancer cells expressed the ADRN marker *SOX11* (Figure 3C) but lacked the expression of MES marker *PRRX1* (Figure 3D). This observation was validated in a second scRNA-Seq dataset (Dong, et al.) (26), where *H2AFY* mRNA was found in ADRN NB cancer cells, but was lowly expressed in cells of the MES lineage (Figure 3E and Supplemental Figure 2, A–C). Moreover, *H2AFY* was expressed by myeloid cells in NB tumor tissues but the expression did not differ among major myeloid cells i.e., macrophages, monocytes, and dendritic cells (DCs), in the 2 patient datasets (Supplemental Figure 2, D and E). Of note, *H2AFY*-high ADRN cancer cells in NB tumors demonstrated a gene expression signature associated with increased proliferative capacity (Supplemental Figure 2F). Therefore, we hypothesized that *H2AFY* may serve as an epigenetic gatekeeper for cell state transition and malignant behavior in human NB.

### H2afy in MYCN-driven NB cells controls the epigenetic and translational landscape.

To reveal the cancer intrinsic network controlled by *H2afy*, we employed a mouse NB cell line, 9464D, which was originally established from spontaneous tumors of the *MYCN*-driven transgenic mouse model (27). Compared with other murine NB cell lines, 9464D cells resemble patients with high-risk disease, because it is dependent on the oncogenic signaling of *MYCN,* and lack surface expression of MHC molecules (Supplemental Figure 6A). *H2afy* was deleted in 9464D cells by transfecting RNP complexes containing the gene-specific crRNA. Control (ctrl) cells were generated at the same time using RNP complexes without the crRNA. Because of its role in chromatin remodeling, we mapped the epigenetic landscape of control and *H2afy* CRISPR-KO cells using ATAC-Seq (28, 29). Analysis of the ATAC-Seq results confirmed the data quality with a FRiP score exceeding 20% (Supplemental Figure 3A and Supplemental Data 2). Although genomic annotation of all peaks (Supplemental Figure 3B) was comparable between KO and control cells, differential peak analysis indicated that epigenetically active sites were preferentially located in the promoter regions of the KO cells (Supplemental Figure 3C).

Genes associated with the MES-like cell state, e.g. *Prrx1, Flrt2,* and *Col5a1,* were more epigenetically active in KO cells, while the chromatin state of ADRN-like genes, e.g., *Sox11, Nefl,* and *Rbms3*, was suppressed (Figure 3, F and G). This observation was confirmed using an extended panel of MES/ADRN genes (30) in control and KO cells (Figure 3H). Moreover, KO cells demonstrated substantially altered transcription factor motifs, e.g., *Stat1, Stat2,* and *Irf/Nfkb,* suggesting enhanced cancer immunogenicity at the epigenetic level (Figure 3I). Pathway enrichment analysis of 805 differentially expressed peaks between KO and control cells revealed enhanced synapse organization (Supplemental Figure 3D) and reduced epithelial cell proliferation (Supplemental Figure 3E) upon *H2afy* deletion.

**Figure 4:**
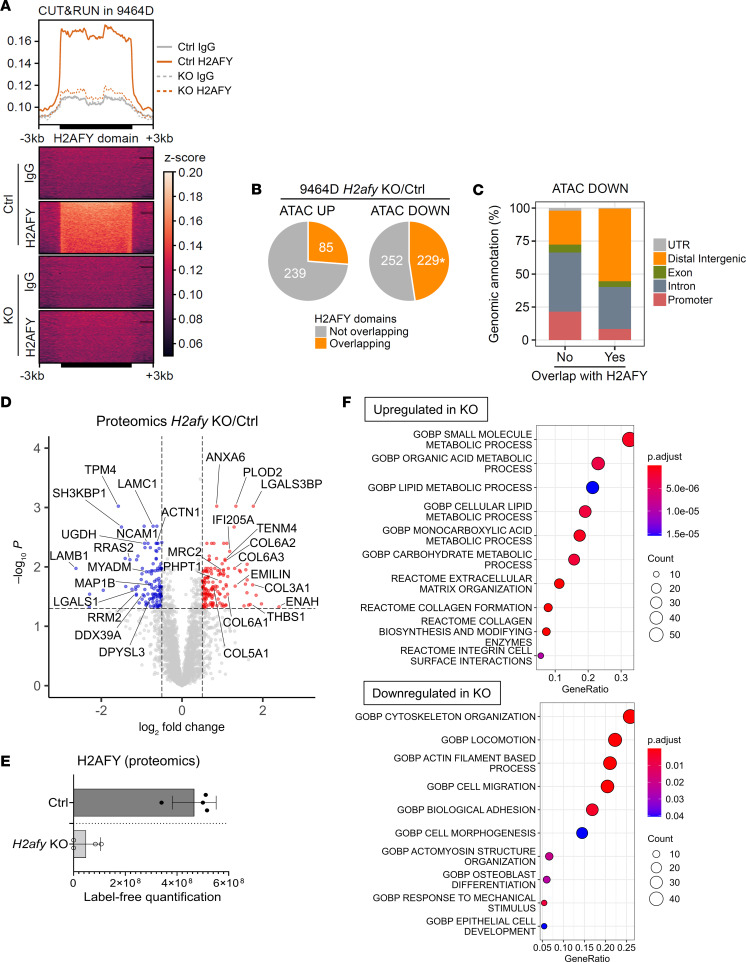
Epigenetic and translational profiling of *H2afy*-deficient NB cells. The regulatory role of H2AY protein in control or KO 9464D cells was mapped using CUT&RUN. (**A**) Heatmap and mean profile visualization of the H2AFY CUT&RUN signal in 9464D cells across enriched domains was identified with epic2, using KO cells as the negative control. Every region was scaled to the same size and extended +/– 3 kb in each side. A nontargeting IgG was used as a negative control. The average signal of 2 experimental replicates is represented. (**B**) Number of overlapping differential ATAC-Seq peaks with H2AFY domains from the CUT&RUN dataset is shown. * *P* < 0.05, permutation test. (**C**) Genomic annotation distribution of downregulated ATAC-Seq peaks classified by their overlap with H2AFY-enriched domains in CUT&RUN. The translational landscape in control or H2AFY-deficient 9464D cells was mapped using label-free proteomics. (**D**) Differentially expressed proteins were visualized in a volcano plot using Log_2_ FC and Log_10_ *P* values. (**E**) The lack of H2AFY protein was confirmed using proteomics. Pathway analysis according to the (**F**) upregulated proteins or downregulated proteins in KO cells compared with control cells (FDR < 0.05 and Log_2_FC > 0.5).

To test for a potential direct role of H2AFY in regulating changes in chromatin accessibility in 9464D cells, we performed CUT&RUN analysis using an established protocol (31). We obtained a specific signal for the genomic distribution of H2AFY in 9464D cells shown by the loss of signal in KO cells (Figure 4A and Supplemental Figure 4A). Domains of H2AFY enrichment overlapped with regions that changed accessibility, as determined by ATAC-seq regions. Yet, this overlap represented a significant and higher-than-randomly expected association only in the case of regions that lost chromatin accessibility (Figure 4B and Supplemental Figure 4B). The genomic annotation of these down-regulated ATAC-Seq peaks in KO cells overlapping with H2AFY showed a higher representation of distal intergenic elements and lower proportion of promoters compared with downregulated regions not overlapping with H2AFY (Figure 4C). Taken together, the genomic localization of H2AFY is directly related to the regulation of distal regulatory elements, which lose accessibility upon deletion of the protein.

To further validate whether the epigenetic changes of MES/ADRN genes in KO cells were relevant at the translational level, we mapped the protein expression landscape in control and *H2afy*-deficient 9464D cells using label-free mass spectrometry. In line with previous results, we observed upregulation of MES-like proteins, e.g., ENAH, COL6A1-3, ANXA6, and COL3A1, but downregulation of ADRN-like proteins, e.g., RRM2, NCAM1, DPYSL3, and DDX39A in KO cells (Figure 4D and Supplemental Data 3). Moreover, deletion of the H2AFY protein was confirmed using proteomics (Figure 4E) but neither SOX11 nor PRRX1 were detected, probably due to the low protein abundance.

Using a defined threshold (FDR < 0.05 and Log_2_FC > 0.5), we performed pathway enrichment analysis and demonstrated upregulation of metabolic processes and downregulation of cytoskeleton organization and cell migration in H2AFY-KO cells (Figure 4F). Protein network analysis using the STRING database and functional enrichment identified 5 key functional groups represented by the differentially expressed proteins, which indicated substantially changed metabolic process, cell cycle, and differentiation in *H2afy*-KO cells (Supplemental Figure 4C). We concluded that H2AFY sustained the ADRN cell state in human NB cells and its removal facilitated transition to a MES-like state.

### Genetic deletion of H2afy in murine NB cells reverts ICB resistance in vivo.

**Figure 5:**
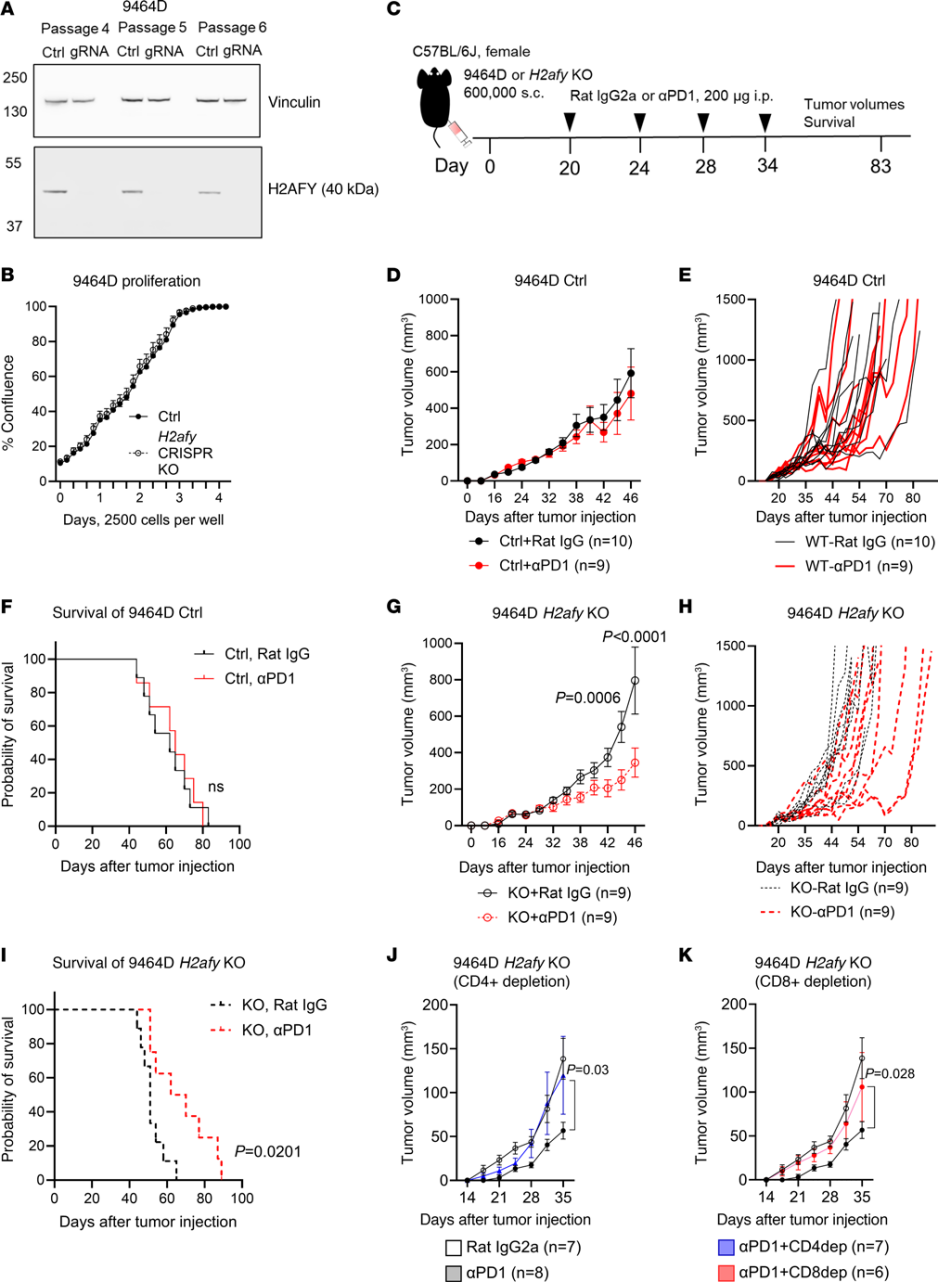
Genetic deletion of *H2afy* in NB cancer cells reverted resistance to PD-1 blockade. (**A**) The *H2afy* gene was targeted by CRISPR/Cas9 in the *MYCN*-driven 9464D cancer cells. Expression of the H2AFY protein was detected using Western blotting at different cell passages. Representative blot of 2 biological repeats. (**B**) Proliferation of control (ctrl) and KO 9464D cells was compared using the Incucyte live-cell imaging system. A representative experiment of 3 biological repeats. (**C**) Treatment schedule of mice bearing ctrl or KO 9464D cells. (**D**–**F**) Comparison of average tumor volumes (mean±SEM), growth of individual tumors and survival between mice bearing s.c. ctrl 9464D tumors that were treated i.p. with a rat IgG2a isotype control (clone 2A3) or an αPD-1 antibody (clone RMP1-14) at 200 μg per mouse, 9 or 10 mice per group. (**G**–**I**) Comparison of average tumor volumes (mean±SEM), growth of individual tumors and survival between mice bearing s.c. *H2afy*-KO 9464D tumors that were treated with the rat IgG isotype or αPD-1 at 200 μg per mouse, 9 or 10 mice per group. 1 day before IgG or α-PD1 treatment, mice bearing *H2afy*-KO 9464D tumors were treated with depletion antibodies against (**J**) CD4^+^ T cells (clone GK1.5) or (**K**) CD8^+^ T cells (clone 2.43) at 100 μg per mouse (i.p.) every 5 days, 6–8 mice per group. Tumor growth was compared among groups using 2-way ANOVA. Survival of mice in different groups was depicted using Kaplan-Meier curves with a Log-rank (Mantel-Cox) test.

Given that NB cells at the MES state were more immunogenic (32, 33), we sought to test whether *H2afy* deficiency could improve antitumor immunity against the immunologically cold 9464D tumors (34–36). Our optimized CRISPR/Cas9 protocol resulted in sustained H2AFY protein (Figure 5A) and mRNA deletion (Supplemental Figure 5A) in 9464D cells without single-cell cloning. Importantly, H2AFY protein expression was stable in control cells and the protein remained absent in KO cells among passages (Figure 5A and Supplemental Figure 5B). Deletion of *H2afy* did not impact its proliferative capacity in cell culture (Figure 5B).

As expected, 9464D tumor-bearing mice were unresponsive to PD-1 blockade therapy (Figure 5, C, D, and E) and the therapy failed to extend the survival of tumor-bearing mice (Figure 5F). *H2afy*-deficient tumors showed comparable growth patterns to the control tumors when treated with an isotype control antibody (Supplemental Figure 5C). In contrast, mice bearing *H2afy*-KO tumors demonstrated significantly delayed tumor growth (Figure 5, G and H) and prolonged survival (Figure 5I) in response to PD-1 blockade therapy. Depletion of CD4^+^ (Figure 5J) or CD8^+^ T cells (Figure 5K) abrogated the superior antitumor efficacy in KO tumors and depletion of NK cells partially compromised the antitumor efficacy (Supplemental Figure 5D). Using a flow cytometry–based protein detection method (Supplemental Figure 5E), KO tumors escaping immune surveillance remained negative for H2AFY protein at the study endpoint (Supplemental Figure 5F).

Phenotypic profiling of 9464D cancer cells in vitro demonstrated the absence of surface MHC-I (H2-Dk/Dd) and MHC-II (I-A/I-E) and a low expression of PD-L1, which were not altered in KO cells (Supplemental Figure 6A). Expression of immune-related markers was further examined using a public scRNA-Seq dataset of the human SK-N-SH cell line that contains 2 distinct ADRN/MES subsets (37). In line with our earlier data, *H2AFY* mRNA was coexpressed with ADRN-like genes, i.e. *SOX11, CD24,* and *PHOX2B*, but was expressed at low levels in the MES subset (Supplemental Figure 6B). *HLA-B*, *HLA-C,* and *PD-L1,* but not *HLA-A*, demonstrated enhanced expression in *H2AFY*-low MES-like cells (Supplemental Figure 6B). However, treatment with recombinant mouse IFNG (Supplemental Figure 6C) or TNFA (Supplemental Figure 6D) failed to impair the proliferation of control or 9464D-KO cells in vitro. Therefore, we concluded that *H2afy* conferred primary immune resistance to ICB therapy in *MYCN*-driven NB and its deletion could potentiate immunogenicity in NB cells.

### Activation of multiple immunological pathways contributes to efficacy against the H2afy-KO NB tumors.

Next, we performed in vivo studies to address local and systemic immunological changes using a nanostring mRNA panel and multicolor flow cytometry (Supplemental Figure 7, A and D). In mice bearing control tumors, PD-1 blockade therapy increased the expression of immune-related genes, e.g. *Cxcr5* and *Il2ra,* but decreased the expression of genes associated to the innate immunity, e.g., *Sirpa, Tlr7,* and *Tlr8* (Supplemental Figure 7B and Supplemental Data 2). Moreover, we validated a number of genes that showed similar patterns at the epigenetic and transcriptional levels when comparing ATAC-Seq from the 9464D cell line pair and mRNA expression data from tumor-bearing mice (Supplemental Figure 7C).

**Figure 6:**
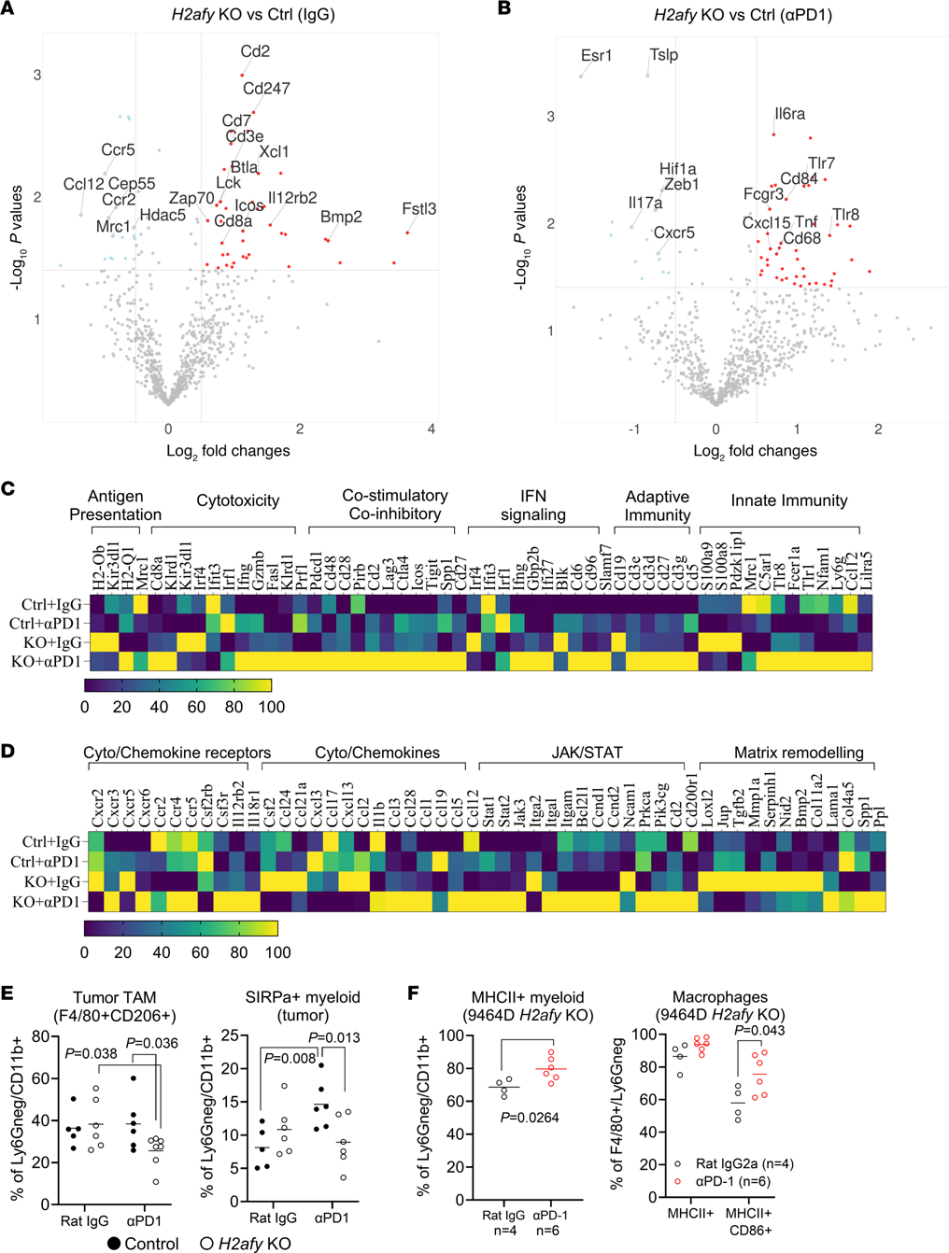
Concurrent activation of adaptive and innate immunity enabled antitumor immunity in *H2afy*-KO tumors. Control (ctrl) or KO 9464D tumors were harvested after the last dose of rat IgG2a isotype control or α-PD-1 antibody. Single cells were generated from tumors and mRNA were isolated for Nanostring analysis. Differentially expressed mRNAs were compared between KO and ctrl mice treated with (**A**) IgG or (**B**) α-PD1, via unpaired 2-tailed *t* test. (**C** and **D**) Genes were grouped according to functions and their expressions were shown for all groups. (**E**) Single cells were generated from mice bearing ctrl or KO 9464D tumors in different treatment groups (5–7 mice per group) and myeloid cells were characterized using flow cytometry. Statistical differences among groups were analyzed using a 2-way ANOVA. (**F**) Single cells from mice bearing KO 9464D tumors treated with a rat IgG isotype control (*n* = 4) or the α-PD-1 antibody (*n* = 6) were isolated and activation of myeloid cells was characterized using flow cytometry, unpaired 2-tailed *t* test.

When evaluating the mRNA expression of *H2afy*-deficient tumors in mice treated with the isotype control antibody (Figure 6A), we observed significantly enriched genes associated with immune infiltration (*Cd8a, Cd3e, Cd2, Cd7,* and *Xcl1*), T cell signaling (*Lck* and *Zap70*) and immune activation (*Cd247, Btla, Icos,* and *Il12rb2*). Meanwhile, mRNA expression associated with the *Ccl12-Ccr2/5* axis was impaired in the *H2afy*-deficient tumors (Figure 6A). Upon the PD-1 blockade therapy, *H2afy*-KO tumors demonstrated stronger expression of genes associated with inflammatory innate immunity, e.g., *Tlr7, Tlr8, Cd68, Cd84, Tnf,* and *Il6ra* (Figure 6B).

To map the immunological landscape upon *H2afy* deletion in NB cells, we grouped mRNA transcripts according to the biological functions. In line with the ICB-resistant feature of 9464D tumors, PD-1 blockade alone generated a marginal increase in genes associated with cytotoxicity, adaptive immunity, and cytokines/chemokines (Figure 6, C and D). Although *H2afy*-deficient tumors grew comparably with the control tumors, we observed substantially enhanced mRNA expression in antigen presentation (*H2-Ob* and *Kir3dl1*), cytokines/chemokines (*Csf2, Ccl2, Cxcl13,* and *Ccl24*), and the MES-like phenotype (*Loxl2, Tgfb2,* and *Serpinh1) (*Figure 6, C and D). *H2afy*-deficient NB tumors treated with PD-1 blockade demonstrated a proinflammatory microenvironment, demonstrated by enhanced expression of pathway genes for cytotoxicity, costimulation, adaptive and innate immunity, cytokines/chemokines, and JAK/STAT signaling (Figure 6, C and D). Of note, unique genes regulating matrix remodeling (*Lama1, Col4a5, Spp1,* and *Ppl*) were upregulated in this group, compared with KO tumors treated with the isotype control (Figure 6D). This demonstrated that deletion of *H2afy* in NB cells led to remodeling of the NB tumor microenvironment.

To further investigate the local and systemic impact of *H2afy* deficiency in tumor-bearing mice, we conducted flow cytometric analysis on cells isolated from tumors and spleens (Supplemental Figure 7D). Four doses of PD-1 blockade or IgG were given in this study due to the slow tumor growth in the KO group (Supplemental Figure 7D). Tumor-infiltrating CD8^+^ T cells were comparable among groups, while CD4^+^ T cells were less abundant in KO tumors treated with PD-1 blockade (Supplemental Figure 7E). We observed a significant reduction of regulatory T cells (CD25^+^FoxP3^+^CD4^+^ T cells) in *H2afy*-deficient tumors (Supplemental Figure 7F). Moreover, suppressive macrophages (F4/80^+^CD206^+^ or SIRPα^+^) were significantly reduced in *H2afy*-deficient tumors treated with ICB compared with treated control tumors (Figure 6E). Meanwhile, inflammatory myeloid cells (MHCII^+^CD11b^+^) and a subset of immune-stimulatory F4/80^+^ macrophages (MHCII^+^CD86^+^, Figure 6F) were elevated by PD-1 blockade in size-matched KO tumors (Supplemental Figure 7G). Although CD8^+^ T cells in KO tumors did not express more IFNG nor CD69 upon PD-1 blockade (Supplemental Figure 8A), surface expression of a late dysfunctional T cell marker, CD38 (38), demonstrated a marked decrease (Supplemental Figure 8B).

Local deletion of *H2afy* in NB tumors induced a systemic change in splenic CD206^+^ dendritic cells and MHCII-negative monocytes (Supplemental Figure 8C). Further, ICB therapy enhanced the frequencies of splenic PD-1^+^CD8^+^ T cells in mice bearing control tumors, which was significantly reduced in ICB-treated KO tumors (Supplemental Figure 8D). Altogether, our data suggested that adaptive and innate immunity collaborated to enable superior tumor control in *H2afy*-deficient NB tumors upon ICB treatment.

### A multiomics approach reveals prognostic genes linked to H2AFY in human NB.

**Figure 7:**
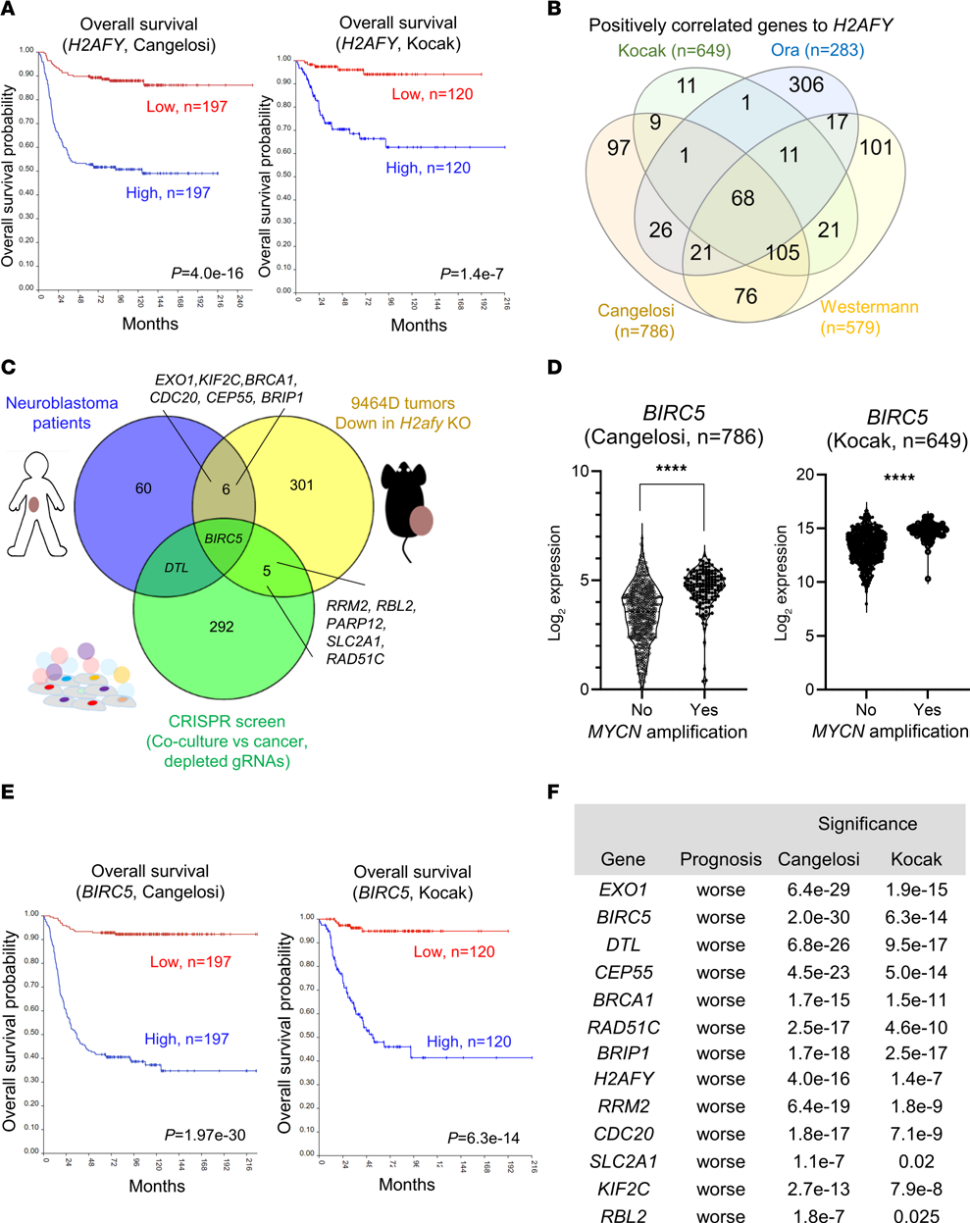
A multiomics approach to identify prognostic genes linked to *H2AFY* in human NB. (**A**) Comparison of overall survival in patients with high or low *H2AFY* mRNA in tumors in 2 independent RNA-Seq datasets (top 25% versus bottom 25%) using Kaplan-Meier curves. (**B**) Overlapping genes that are positively correlated with *H2AFY* mRNA in 4 large datasets of patients with NB. (**C**) Prioritization of genes linked to *H2AFY* by overlapping hits from experimental and clinical datasets**.** (**D**) Expression of *BIRC5* mRNA in *MYCN*-amplified and nonamplified patients with NB. (**E**) Prognostic value of *BIRC5* mRNA in 2 independent cohorts of patients with NB (top 25% versus bottom 25%) using Kaplan-Meier curves. (**F**) The prognostic value of overlapping genes from at least 2 different datasets (top 25% versus bottom 25%).

To examine the prognostic values of *H2AFY* mRNA in human NB, we employed public bulk RNA-Seq datasets from the R2: Genomics Analysis and Visualization Platform (http://r2.amc.nl http://r2platform.com). Using 2 large datasets of patients with NB (39, 40), we showed that low *H2AFY* mRNA expression significantly correlated with favorable overall survival in patients with NB (Figure 7A). Because high *H2AFY* mRNA expression is associated with a more proliferative cancer phenotype (Supplemental Figure 2F), we tested its prognostic value independent of *MYCN* amplification. Using age as a clinical parameter, we showed that high *H2AFY* mRNA was associated with worse survival in both low-risk (< 18 months, Supplemental Figure 8E) and high-risk (> 18 months, Supplemental Figure 8F) patients. Moreover, high *H2AFY* mRNA was associated with worse overall survival in low-risk patients without *MYCN* amplification (Supplemental Figure 8G). Therefore, we propose that *H2AFY* expression is a *MYCN*-independent prognostic marker.

Although direct targeting of H2AFY remains difficult, a histone deacetylase (HDAC) inhibitors, such as sodium phenylbutyrate (SPB), downregulated the expression of *H2AFY* mRNA in patients with Huntington’s disease (41). However, HDAC inhibitors under clinical testing, i.e. SPB, Entinostat or RG2833, failed to suppress H2AFY expression in 9464D NB cells in vitro (Supplemental Figure 8H). Therefore, we sought to identify genes associated with *H2AFY* mRNA in NB tumors to reveal alternative targets. We included 2 additional datasets from Ora et al. (42) and Westermann et al. (R2 identifier: ps_avgpres_nbsewester579_gencode19) and extracted 68 common genes that positively correlated with *H2AFY* mRNA using a cutoff of R^2^ > 0.5 (Figure 7B and Supplemental Data 5).

To examine the functional causality of genes associated with *H2AFY*, we leveraged our unique multiomics datasets generated in this study, which combined mRNA analysis of tumor-bearing mice, genome-wide CRISPR screens using TICS and public transcriptomics data from patients with NB (Figure 7C). Despite using datasets from distinct experimental settings and host species, we uncovered overlapping genes among datasets (Figure 7C). In particular, our combined analysis uncovered that *BIRC5* was strongly associated to *H2AFY* in patients with NB, downregulated in mice bearing *H2afy*-KO tumors, and was among the top depleted genes in the CRISPR screen (Figure 7C). Moreover, we identified additional common genes in at least 2 datasets. These included *DTL* (patients versus CRISPR screen) and *EXO1, KIF2C, BRCA1,* *CDC20, CEP55,* and *BRIP1* (patients versus mice), and *RRM2, RBL2, PARP12, SLC2A1,* and *RAD51C* (mice versus CRISPR screen) (Figure 7C).

Validation analysis in datasets from patients with NB confirmed that *BIRC5* mRNA was expressed at a significantly higher level in *MYCN*-amplified tumors (Figure 7D) and strongly predicted patient survival (Figure 7E). Moreover, all overlapping genes, except *RBL2*, demonstrated statistically significant prognostic values in 2 cohorts of patients with NB (Figure 7F). Together, we have utilized a unique multiomics approach to verify the clinical relevance of H2AFY in patients with NB and revealed putative drug targets to improve the immunogenicity of NB cells.

## Discussion

Eliciting immune responses against human cancers has brought substantial clinical benefits to patients. However, high-risk NB with amplification of the *MYCN* oncogene presents a therapy-resistant phenotype with low mutational burden (10, 11) and poor expression of immunological receptors (14, 43, 44). We characterized a panel of human NB cell lines and detected surface HLA class I molecules on 5 out of 6 *MYCN*-amplified cell lines. In contrast, expression of surface PD-L1 was absent on all NB cell lines regardless of *MYCN* status. Human NB cell lines demonstrated a clear response to IFNG stimulation and the induction of HLA-ABC, and the expression of HLA-ABC and PD-L1 showed a strong correlation after IFNG treatment. This is in line with previous reports, where *MYCN*-amplified human NB cell lines exhibited an intact JAK/STAT signaling cascade (32) and could upregulate surface HLA-ABC and PD-L1 in response to IFNG stimulation (43, 45). Therefore, we believe that, besides the *MYCN* oncogene, certain other pathways, e.g., *c-Myc* (14), are at play in repressing immunogenicity in human NB cells.

Utilizing genome-wide CRISPR/Cas9 screens in a human coculture assay, we revealed the previously undescribed function of epigenetic regulator H2AFY as a resistance mechanism to PD-1 blockade in high-risk NB. H2AFY belongs to the macroH2A variants and is part of the nucleosome that prevents transcription factor binding and hampers SWI/SNF nucleosome remodeling (46). These variants have been shown to regulate cell plasticity and act as a barrier for cell reprogramming toward pluripotency (47, 48) and cancer cell stemness (49). Although the precise molecular mechanism of H2AFY function remains elusive, our data is in line with previous studies suggesting that H2AFY might exert its function through regulating the openness and 3-dimensional chromatin structure of distal regulatory elements such as enhancers (50–52). Genetic deletion of the *H2afy* gene using CRISPR/Cas9 neither reduced 9464D cell proliferation in vitro nor tumor growth in immune competent mice, but reverted in vivo resistance to PD-1 blockade (34, 35, 53). Pathways associated with effective immunotherapy, such as infiltration of T cells, JAK/STAT, cytotoxicity, and proinflammatory cytokines, were strongly upregulated in KO tumors.

We observed a favorable balance between myeloid cells with stimulatory and suppressive phenotype in *H2afy*-deficient tumors. We showed previously that NB tumors recruited suppressive myeloid cells (54, 55) and inhibition of these cells synergized with PD-1 blockade against *MYCN*-driven tumors in spontaneous (54–56) and transplantable mouse models (34). It is worth noting that in hepatoblastoma cells, H2AFY altered the response to different cytokines that are produced by myeloid cells (51). In melanoma, H2AFY modulated the tumor immune microenvironment by suppressing inflammatory gene expression in tumor-associated fibroblasts (50). Therefore, it can be speculated that *H2afy* deletion alters the interplay between NB and myeloid cells, leading to a proinflammatory milieu.

Recent evidence demonstrates that the heterogeneity of NB cells is defined by 2 epigenetic states, namely the MES and ADRN lineages (30, 57). Emerging results support that NB tumors in the MES state present a proinflammatory phenotype and are more sensitive to ICB therapy (32, 33). Importantly, our analysis of scRNA-Seq data from NB tumors and the SK-N-SH cell line showed a robust *H2AFY* expression in ADRN-like cancer cells. Mechanistic validation using ATAC-Seq in the KO/ctrl cell line pair revealed enhanced chromatin accessibility for the MES-like signature gene *Prrx1* (58) in KO cells. Conversely, the ADRN signature gene *Sox11* (59) demonstrated reduced chromatin accessibility upon *H2afy* deletion. The epigenetic activity of transcription factor motifs linked to cancer immunogenicity, e.g., *Irf1/2/8/9, Stat1,* and *Nfkb1*, were increased in KO cells, which coincided with findings in a PRRX1-overexpressing cell line model (33).

In the current study, we analyzed the expression of 800 selected immune-related genes in tumor-bearing mice. Of interest, *H2afy*-deficient tumors substantially increased the expression of MES-like genes (*Tgfb2, Loxl2,* and *Serpinh1*), which diminished upon treatment with the PD-1 blocking antibody. These observations suggest that the cell state switch is sustained in vivo and MES-like cells could be preferentially eliminated by the immune system upon ICB therapy due to increased immunogenicity (32, 33). Given that the intrinsic plasticity of epigenetic cell state in NB is modulated by external factors (37), it would be worthwhile to investigate whether H2AFY expression in NB cells can be regulated by external stimuli. Further studies using scRNA-Seq are warranted to elucidate how the ADRN/MES cell state orchestrates the interplay between NB cells and other cell types or stimuli in NB mouse models.

Targeting epigenetic circuits has demonstrated clinical efficacy in treating human cancers (60), including NB (61). The immune modulatory role of these compounds has also been investigated. For example, the FDA-approved inhibitor against HDAC1/3, entinostat, enhances NB immunogenicity by inducing an MES-like phenotype (62). Moreover, HDAC inhibition was shown to reduce the expression of *H2AFY* mRNA in mice and humans (41). However, these compounds failed to directly suppress H2AFY protein expression in 9464D cells. Because it remains challenging to target H2AFY, we leveraged our unique datasets across species and identified a strong link between *BIRC5* and *H2AFY*. The *BIRC5* gene encodes survivin, which is an antiapoptotic protein and has been extensively studied as a therapeutic cancer target (63). Therefore, the mechanistic link between H2AFY and BIRC5, as well as other known epigenetic regulators, should be further characterized to design optimal epiimmunotherapy against high-risk NB.

The epigenetic cell state of NB cells is linked to sensitivity to treatments. On the one hand, MES-like NB cells confer resistance to chemotherapy (30), ALK inhibition (64), and anti-GD2 antibodies (65). On the other hand, NB cells in this state demonstrate a more inflammatory phenotype (32) and are more amenable to immune-mediated cytotoxicity (33). Our work demonstrates that transition to a MES-like state upon H2AFY deletion in ADRN NB cells reverts resistance to ICB immunotherapy. This argues that H2AFY inhibition in combination with chemoimmunotherapy could be more efficacious in preventing disease relapse in patients with NB by simultaneously targeting cancer cells in both epigenetic states.

## Methods

Details of the antibodies (Supplemental Table 1), reagents (Supplemental Table 2), crRNA or primer sequences (Supplemental Table 3) used are summarized in the Supplemental Tables.

### Sex as a biological variable.

Our study utilized only female mice due to slow growth of the tumor model and aggressive behavior of the male mice in this strain, which would not allow successful completion of the studies. It is unknown whether the findings are relevant for male mice.

### Cell culture.

Human NB cell lines were gifted by Christer Einvik (UiT The Arctic University of Norway, Trömso, Norway). Murine NB cell line 9464D was initially established in C57BL/6 transgenic mice that spontaneously overexpressed TH-*MYCN* (66) and was a gift from Malin Wickstörm (Karolinska Institutet, Solna, Sweden). All cell lines were cultured at 37°C with 5% CO_2_ using IMDM (Thermo Fisher Scientific) supplemented with 10% heat-inactivated FBS and 1% Penicillin-Streptomycin (Thermo Fisher Scientific). Cell lines were routinely assessed for mycoplasma infection (MycoAlert, Lonza) and authenticated by DNA fingerprinting (Eurofins).

### Isolation of lymphocytes.

Buffy coats from anonymous healthy individuals were collected from Uppsala University Hospital for isolating peripheral blood mononuclear cells (PBMCs). Blood was carefully laid over 15 mL of LymphoPrep solution in SepMate tubes (StemCell Technologies), followed by centrifugation at 1,200*g* for 10 minutes. Cells were harvested and washed twice with 35 mL PBS and treated with RBC lysis buffer (Biolegend) at room temperature in the dark for 10 minutes. Next, lymphocytes were enriched by eliminating primary monocytes from PBMCs using EasySep CD14^+^ selection kit (StemCell Technologies) according to the manufacturer’s protocol. The isolated primary lymphocytes were either used on the same day or stored in ultra-low temperature freezers.

### TICS.

For setting up TICS, NB cells were harvested and plated onto 96-well flat bottom plates in 100 μL cell culture medium and incubated overnight at 37°C. The following day, healthy donor-derived lymphocytes were counted and labelled with a Cell Tracer Violet (CTV) dye (Thermo Fisher Scientific) in the dark for 10 minutes. After washing with PBS, lymphocytes were resuspended at 3 × 10^6^ cells per mL and added to cancer cells in 100 μL of culture medium, with or without 10 μg/mL of nivolumab (Bristol-Myers Squibb) or durvalumab (AstraZeneca). After 5 days of coculture, secretion of cytokines such as IFNG and granzyme B were measured by ELISA (MabTech) using supernatants harvested from the cocultures. Further proliferation and expression of surface proteins on immune cells were determined by flow cytometry.

### Whole-genome CRISPR screens in TICS.

Genome-wide CRISPR screens of the human NB cell line IMR32 were performed and analyzed using TICS according to a published procedure (18). The *H2afy* gene was deleted in murine NB cell line, 9464D, by transfecting ribonucleoprotein complexes, according to a previous study (18). More information can be found in Supplemental Methods.

### Western blotting.

To determine the expressions of individual proteins, cells were lysed for 15 minutes at 4°C in the RIPA buffer (Thermo Fisher Scientific) supplemented with 10% protease inhibitor cocktail (Thermo Fisher Scientific) before centrifugation at 17,000*g* for 10 minutes at 4°C. Supernatants were quantified using a Bicinchoninic Acid (BCA) Assay (Thermo Fisher Scientific) and stored in a –20°C freezer. Next, lysates were denatured for 12 minutes at 70°C with 4 × SDS loading dye. Protein lysates were loaded onto 4%–12% precast Bis-Tris gels (Invitrogen) for PAGE and transferred onto a nitrocellulose membrane using iBlot system (Invitrogen). Membranes were blocked for 1 hour with 5% skimmed milk blocking buffer before overnight incubation with primary antibody at 4°C. Next, membranes were incubated for 1 hour with appropriate HRP-conjugated secondary antibody and incubated in the substrate solution for protein visualization using an Amersham Imaging system (GE Healthcare).

In some experiments, 9464D cells (5 × 10^5^) were seeded in a 6-well plate and HDAC inhibitors, i.e., Entinostat (Selleck Chemicals), SPB (Selleck Chemicals), or RG-2833 (MedChem Express) were added at 5 or 10 μM in 0.1% DMSO after 24 hours. DMSO alone was used as a control. Cells were cultured for an additional 48 hours and the expression of H2AFY was measured using Western blotting.

### NB mouse tumor model.

Female C57BL/6J mice (8–10 weeks old, purchased from the Charles River Laboratories) were used to establish NB tumor model by s.c. injection of 9464D cells (6 × 10^5^ per mouse). When 80% of the mice developed palpable tumors, mice were treated i.p. with either an anti-PD-1 (α-PD-1) (antibody (clone RMP1-14) or a rat IgG2a isotype control, 200 μg per mouse every 4 days. For the immune depletion study, 100 μg of anti-CD4 (Bio-X-Cell), anti-CD8α and anti-NK1.1 antibodies were i.p. injected per mouse in 100 μL PBS 1 day before the initiation of immunotherapy and continued once every 6 days. Tumor length and width were measured using a digital caliper and tumor volumes were calculated using the formula (length × width^2^)/2 until the maximum humane endpoint of 1.5 cm^3^.

At the end of the study, tumors were harvested and single cells were isolated with the GentleMacs device using a tumor dissociation kit (Miltenyi Biotech). Splenocytes were isolated by passing spleens through 40 μm cell strainers, followed by incubation with the RBC lysis buffer (BioLegend) for 3 minutes on ice and washed with PBS. The single-cell suspensions were either analyzed the same day or stored at –80°C for subsequent use.

### Proteomics and data analysis.

The translational landscape of 9464D control and *H2afy*-KO cells were analyzed using label-free mass spectrometry as previously described (18). Detailed experimental procedure and data analysis pipeline can be found in Supplemental Methods.

### Nanostring analysis and real-time PCR.

To determine gene expression*,* mRNA were isolated from cells using the RNeasy Mini Kit (Qiagen) according to the manufacturer’s protocol. After isolation, the purity and the concentration of the samples were determined using Nanodrop spectrophotometer (Thermo Fisher Scientific).

Gene expression in tumor samples were analyzed using nCounter technology. In brief, mRNA was extracted from single cells isolated from mouse tumors using RNeasy kits (Qiagen) according to the manufacturer’s protocol. mRNA per sample was quantified using NanoDrop 2000 spectrophotometer to provide 100 ng mRNA at the concentration of 20 ng/μL. Samples were analyzed at KI gene facility using a robust gene expression analysis system by multiplexing mRNA samples to up to 800 gene targets from nCounter PanCancer IO 360 Panel.

To determine the expression of *H2afy* in 9464D cells, first-strand complementary DNA (cDNA) was synthesized with 2 μg of RNA using iScript cDNA synthesis kit (BioRad) according to the manufacturer’s instructions. The cDNA templates were used to quantify *H2afy* mRNA expressions on the StepOne Plus system (Thermo Fisher Scientific), with β-actin as a reference gene. Changes in mRNA expression were calculated using 2^–ΔΔCt^ values that were normalized between test and house-keeping control samples.

### Flow cytometry.

For in vitro experiments, untreated 9464D cells or human NB cells were cultured with or without 50 ng/ml rhIFNG (Peprotech) for 16-18 hours. Cells were harvested by gentle cell scraping and surface markers were stained using a panel of FACS antibodies.

For in vivo studies, spleens and tumors were harvested from the experimental mice to obtain splenocytes and single cells as described above. Cells were seeded in a 96 well v-bottom plate and stained with a mixture of an Aqua Fixable Live/Dead maker (1:200, Invitrogen) and an anti-mouse CD16/32 antibody (1:100, Invitrogen). After washing with PBS, cells were stained with a panel of fluorochrome-conjugated antibodies for surface proteins for 30 minutes at 4°C, followed by cell fixation and permeabilization using either the FOXP3 buffer set (Invitrogen) or the True-Nuclear buffer set (Biolegend), according to manufacturer’s instructions. Next, fluorochrome-conjugated antibodies (1:50) were incubated with cells in order to detect intracellular proteins. In some experiments, the anti-H2AFY antibody (Abcam) was conjugated with a Zenon labelling kit for rabbit IgG (Invitrogen) and added at 5 ng/mL per well after cell fixation and permeabilization, in order to detect the intracellular expression of H2AFY. Antibody-stained samples were quantified using a BD Fortessa (BD Bioscience), a CytoFLEX S, or LX flow cytometer (Beckman Coulter).

### Live cell imaging.

The proliferation of cancer cells were monitored using an Incucyte Zoom instrument (Sartorius). Cells were plated at different cell densities in a 96-well flat bottom plate in 100 μL of culture medium. The cell confluence was plotted against time at defined time-intervals to obtain growth rates. In some experiments, control or *H2afy*-KO 9464D cells were seeded in a 24 well plate (5 × 10^4^ cells per well) and recombinant mouse IFNG or TNFA were added after 24 hours at 5 or 50 ng/mL. Cells cultured without cytokines were used as controls. Cell proliferation was recorded for up to 7 days.

### Library preparation for ATAC-Seq.

Standard ATAC-Seq libraries were prepared following the previously established protocol (29). In brief, 5 × 10^4^ mouse NB cells were centrifuged at 500*g* for 5 minutes at room temperature for each reaction. The cell pellet was resuspended in 50 μL lysis buffer containing 10 mM Tris-Cl at pH 7.4 (Invitrogen), 10 mM NaCl (Invitrogen), 3 mM MgCl2 (Invitrogen), and 0.1% IGEPAL CA- 630 (Sigma-Aldrich) and centrifuged at 500*g* for 10 minutes at 4°C. After centrifugation, the cell pellet was immediately processed to transposition reaction and was resuspended in 50 μL transposase mixture containing 25 μL 2 × TD buffer (20 mM Tris-HCl at pH 7.6, 10 mM MgCl2 and 20% dimethyl formamide), 22.5 μL Nuclease-free water (Invitrogen), and 2.5 μL Tn5 transposase, followed by incubation for 30 minutes at 37°C. After the transposition, the samples were purified using Qiagen MinElute PCR Purification kit (Qiagen). The transposed DNA was amplified using NEBNext High-Fidelity 2 × PCR master mix (New England Biolabs), and 1.25 μM of custom Nextera PCR primers 1 and 2 with following this thermal condition; 1 cycle of 72°C for 5 minutes; 98°C for 30 seconds; and 5 cycles of 98°C for 10 seconds, 63°C for 30 seconds and 72°C for 1 minute. qPCR was performed to determine the optimal number of cycles for final PCR amplification. For this, 5 μL of the previously PCR amplified DNA was mixed with 10 μL of the PCR cocktail with SYBR Green at a final concentration of 0.6 × and run on a qPCR machine with the following program: 1 cycle of 98°C for 30 seconds; and twenty cycles of 98°C for 10 seconds, 63°C for 30 seconds and 72°C for 1 minute. The additional cycles needed for the remaining 45 μL of previously PCR amplified DNA was determined by the cycle number at which the fluorescent intensity reached 1/3 of its maximum value in the linear RN-versus-cycle plot. The remaining DNA was PCR amplified using the cycle number determined by qPCR with the following program: 1 cycle of 98°C for 30 seconds; and N cycles (determined by qPCR) of 98°C for 10 seconds, 63°C for 30 seconds and 72°C for 1 minute. The PCR product was purified using Qiagen MinElute PCR Purification kit (Qiagen), followed by a size selection step with SPRI beads with 1:1.2 ratio (Beckman Coulter). Finally, the purified DNA was eluted in 20 μL of Elution Buffer (10 mM Tris-HCl, PH 8).

### ATAC-Seq data processing.

ATAC-Seq sequencing reads (GSE235736) were processed with same pipeline described below. Sequencing adaptor was trimmed by using pyadapter_trim.py (https://github.com/TheJacksonLaboratory/ATAC-seq/blob/master/auyar/pyadapter_trim.py). The sequencing reads were aligned to the reference genome (mm10) using Bowtie 2 (67) with the ‘-very-sensitive’ parameter. The aligned BAM files were sorted and filtered using Samtools (68). PCR duplicates were removed with Picard (http://broadinstitute.github.io/picard/). BigWig files were generated using the ‘bamCoverage’ function in Deeptools (69), with the ‘-normalize Using CPM’ option. The transcription start site (TSS) enrichment score was analyzed using the ‘computeMatrix’ and ‘plotProfile’ functions in Deeptools (70), based on the BAM file. MACS2 (71) was used for peak calling, with the parameter ‘-q 0.01 -nomodel -shift 0’. Mouse blacklist regions were removed using bedtools intersect6. The read counts matrix was generated using the ‘multicov’ function in bedtools intersect (72) and normalized by EdgeR’s ‘cpm’ function (73). A heatmap of Pearson correlation among replicates was visualized using the R package ‘pheatmap’. Differential peak analysis was performed using DeSeq2 (74) with criteria of log_2_(FC) > 1 and FDR < 0.05. A volcano plot of differential peaks was generated using the R package ‘ggplot’. Gene annotation and genomic feature plots were conducted with the R package ‘ChIPSeeker’ (75). Transcription factors were identified using Homer’s ‘findmotifsGenome.pl’ function (76). Enriched TF motifs were analyzed with the R package ‘chromVar’ (77) and visualized using ‘pheatmap’. Gene ontology enrichment analysis was conducted using the R package ‘clusterProfiler’ (78). Sequencing coverage was visualized using the Integrative Genomics Viewer (IGV) (79).

### Library preparation for CUT&RUN.

CUT&RUN reactions were performed as described in Meers et al. (31), following the “Standard CUT&RUN” protocol. Briefly, freshly harvested 9464D cells (1 × 10^6^) were bound to concanavalin-A paramagnetic beads (Epicypher), then split equally, resuspended in antibody binding buffer, and incubated overnight with either home-made macroH2A1 antibodies (80, 81) or an IgG nontargeting control (Abcam ab46540). Both antibodies were diluted 1:100 in the binding reaction. Samples were then washed and bound with pA/G-MNase (Epicypher), and chromatin digestion was started by the addition of CaCl_2_ and stopped after 30 minutes with STOP buffer containing chelating agents. Samples were then incubated for 30 minutes at 37°C to release CUT&RUN fragments and incubated for 1 hour at 50°C with proteinase K, followed by a purification step using ChIP DNA Clean & Concentrator (Zymo Research). Sequencing libraries were prepared with the KAPA HyperPrep kit (Roche) and NEXTflex DNA barcodes for Illumina (Bioo Scientific), quantified with the KAPA Library Quantification kit (Roche), pooled at approximately equimolar concentration and sequenced at Novogene (UK) Co Ltd. in an Illumina NovaSeq instrument to achieve a depth of at least 10 M paired-end 150 bp reads per sample.

### CUT&RUN data processing.

Paired end reads were adapter and quality trimmed with trimgalore using —stringency 3 and aligned using Bowtie2 (82) to the mm10 mouse genome assembly with the following options: —very-sensitive —no-discordant —no-mixed –X 700 –dovetail. The resulting alignment bam files were filtered to retain only concordant proper pair alignments using samtools sam flag 0x2 and minimum mapping quality score of 30. Coverage signal profiles in bigwig format were generated using the bamCoverage function from deepTools (69) with a Counts-Per-Million per-sample normalization using a bin of 100 bp, ignoring ChrM for normalization. These profiles were used for visualization using deepTools computeMatrix and plotHeatmap functions.

Epic2 (83) was used to perform peak calling in the form of broad domain detection on the filtered aligned reads using the KO samples as background, a bin size of 2,000 bp and the following options: —guess-bampe -kd -fdr 0.00001 –gaps-allowed 5. Problematic regions from the ENCODE blacklist were subtracted and domains with a 75% overlap with a blacklisted region were excluded (84). Permutation tests were performed using the regioneReloaded R package using the resampleRegions randomization function with a resampling universe composed of all detected ATAC-Seq peaks (85, 86).

### Analysis of scRNA-Seq data from patients with NB.

Previously published scRNA-Seq data generated by us (25) as well as others (26) were analyzed for this study. For the Dong et al. dataset, raw scRNA-Seq.fastq files were downloaded from the Gene Expression Omnibus (GEO) repository (GSE137804). Files from both datasets were aligned to the GRCh38 genome using 10× Genomics Cell Ranger 7.0.0. Filtered gene expression matrices (from *cellranger* output) were used for subsequent analyses. In the initial filtering step, cells with < 200 expressed genes and < 500 UMIs were discarded. Next, we filtered cells based on the proportion of mitochondrial reads (%mito) and total number of unique genes expressed (nFeature) on a sample-to-sample basis. Cells with %mito more than 2 SDs above mean were removed. Cells with nFeature less than 2 SDs below mean (log_10_-transformed) were removed. Doublets were identified and removed using DoubletFinder v2.0.3 with SCT normalization (87).

After initial QC and doublet removal, Seurat v5 (88) was used for all downstream analyses. In a joint Seurat object, 1 per dataset, raw counts from each individual sample was kept as a layer. After *NormalizeData, FindVariableFeatures, ScaleData,* *RunPCA, FindNeighbors*, and *FindClusters* steps, *RunUMAP* was run with *dims* parameter set to 1:30. Layers were then integrated using *IntegrateLayers* with the *method* parameter set to “HarmonyIntegration”, followed by *JoinLayers, FindNeighbors, FindClusters,* and *RunUMAP*. Markers for each cluster were identified using *FindAllMarkers*. We first annotated the dataset from Olsen et al. on the basis of expression of canonical cell type markers as previously described (25, 89). The Dong et al. dataset was annotated by using the *singleR* R package (90) with the annotated Olsen dataset as reference.

### Data availability.

Raw sequencing results associated with the CRISPR screens are available at Gene Expression Omnibus under accession numbers under accession number GSE275390 and gRNA counts from the CRISPR screens were available in Supplemental Data 1. Raw data from ATAC-Seq and CUT&RUN of control and *H2afy*-KO 9464D cells are available at Gene Expression Omnibus under accession numbers GSE235736 and GSE270196, respectively. Processed data of ATAC-Seq is also provided in Supplemental Data 2. Data from the label-free mass spectrometry of control and *H2afy*-KO 9464D cells is available as Supplemental Data 3. Normalized mRNA counts of in vivo tumor samples are available as Supplemental Data 4. A list of the 68 overlapping genes that associated with *H2AFY* in human NB tumors is provided as Supplemental Data 5. Values of all data points in graphs are reported in the Supporting Data Values file.

### Statistics.

Experimental data were summarized and visualized using the Graphpad Prism software (Dotmatics). Flow cytometry data was analyzed using the Flowjo software (Treestar). Unless otherwise stated, statistical differences were tested using an unpaired 2-tailed *t* test or a 2-way ANOVA for multiple comparisons. The difference in the Kaplan-Meier curves were demonstrated using Log-Rank *P* values. A *P* value less than 0.05 was considered significant.

### Study approval.

All animals were maintained under germ-free condition at the facility in the Rudbeck Laboratory at Uppsala University, Sweden under an approved ethical permit (Dnr: 5.8.18-06394/2020) by the Swedish Board of Agriculture at Jönköping, Sweden.

## Author contributions

YM and DN initiated the study and designed the experiments. DN completed in vitro and in vivo experiments for the manuscript. RTP performed in vitro and in vivo experiments during the revision. DC and MB designed and performed experiments using the CUT&RUN technology and completed the data analysis. MS and DN generated biological samples for the ATAC-Seq experiment. MX performed data analysis for the ATAC-Seq in collaboration with DN. XC supervised the ATAC-Seq study. GK performed the analysis of CRISPR screens in collaboration with MPM. TKO performed the analysis of scRNA-Seq datasets in collaboration with NB. MRB analyzed the proteomics dataset in collaboration with YM. All authors contributed to the writing and revision of the manuscript.

## Supplementary Material

Supplemental data

Supplemental data set 1

Supplemental data set 2

Supplemental data set 3

Supplemental data set 4

Supplemental data set 5

Unedited blot and gel images

Supporting data values

## Version 1 09/10/2024

In-Press Preview

## Version 2 11/01/2024

Electronic publication
