# Supplementary material for: Epigenetic regulation of cell state by H2AFY governs immunogenicity in high-risk neuroblastoma
Source: J Clin Invest. 2024 Sep 10;134(21):e175310. doi: 10.1172/JCI175310 (PMC11527455; doi:10.1172/JCI175310)
Supplement: Supplemental data [file jci-134-175310-s041.pdf]

Supplementary figures and legends

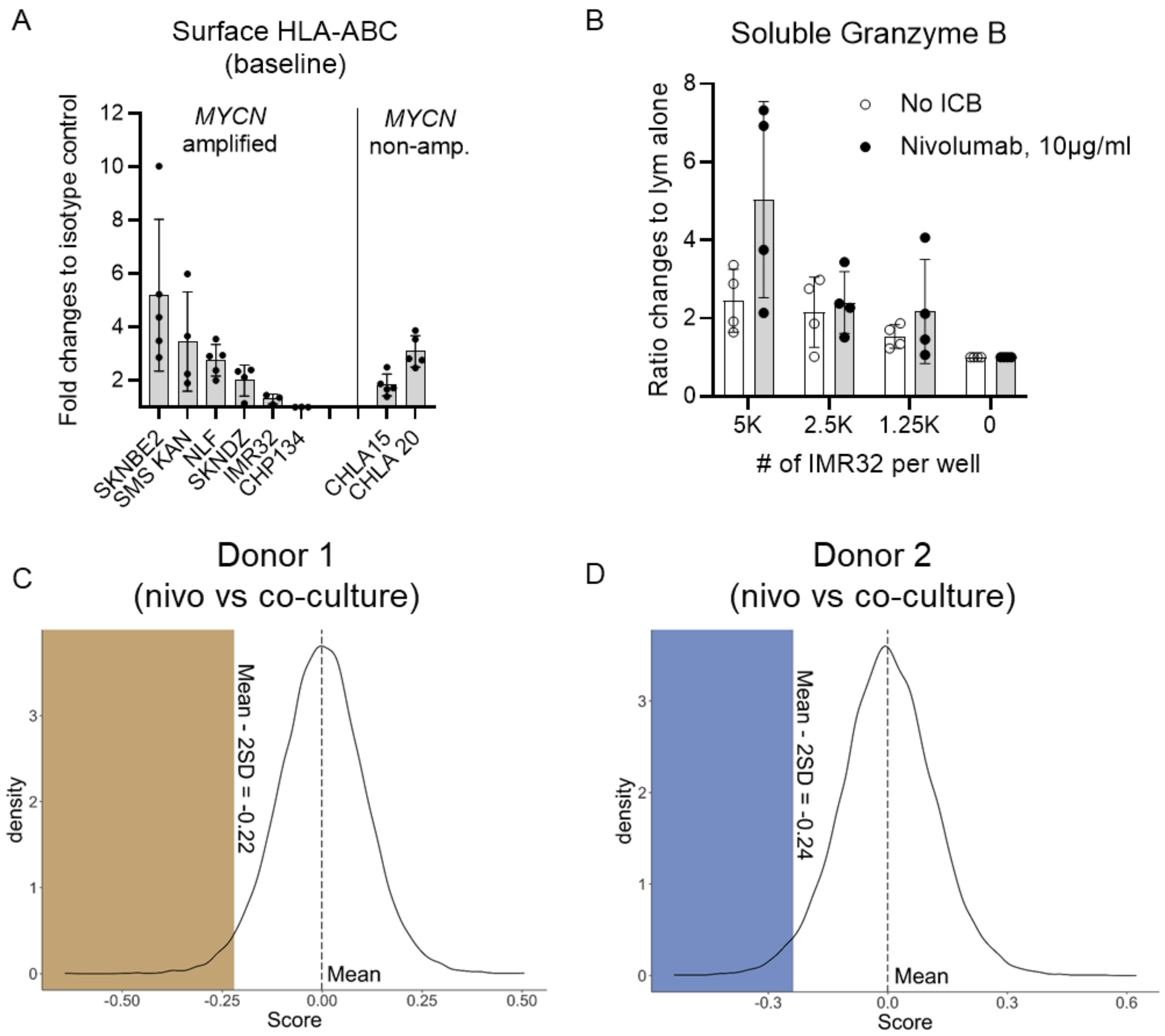

Figure S1. A) Expression of HLA-ABC on human NB cell lines without IFNG treatment. Data was visualized using fold changes to the isotype control antibody as mean±SD. Each dot represented an individual experiment (n=5). **B)** Levels of soluble granzyme B in TICS with IMR32 cells +/- 10 µg/ml nivolumab. Data was normalized to the wells containing only lymphocytes and shown as mean±SD**.** Each dot represented an individual lymphocyte donor (n=4). **C)** and **D)** Distribution of genome-wide gRNA scores from the two independent genome-wide CRISPR screens when comparing nivolumab-treated and non-treated co-cultures. A cut-off of mean minus 2SD was used to select top-depleted genes.

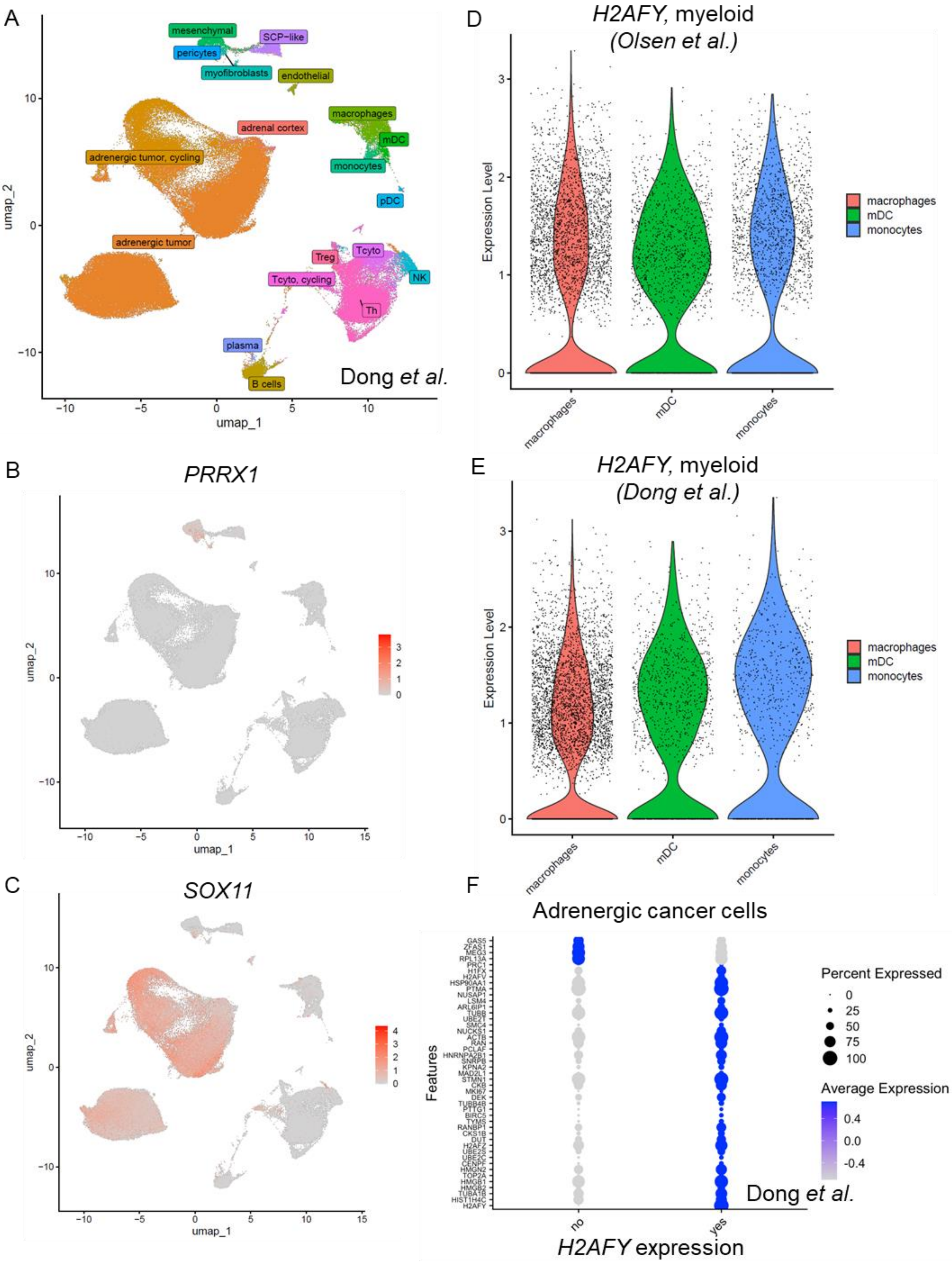

A
mesenchymal
SCP−like
pericytes
myofibroblasts
endothelial
macrophages
adrenal cortex
mDC
adrenergic tumor, cycling
monocytes
pDC
adrenergic tumor
Tcyto
Treg
Tcyto, cycling
NK
Th
plasma
B cells
Dong et al.
umap_1
umap_2
B
PRRX1
C
SOX11
D
H2AFY, myeloid
(Olsen et al.)
Expression Level
macrophages
mDC
monocytes
E
H2AFY, myeloid
(Dong et al.)
F
Adrenergic cancer cells
Features
Percent Expressed
Average Expression
Dong et al.
no
yes
H2AFY expression

Figure S2. A) Analysis and annotation of a scRNA-seq dataset (Dong et al.). Expression of **B)** *PRRX1* and **C)** *SOX11* at the single cell level in different cell subsets. **D-E)** Expression of *H2AFY* mRNA at the single cell level was compared among macrophages, mature dendritic cells (mDC) and monocytes in the two scRNA-seq datasets. **F)** Enriched genes in *H2AFY* high and low adrenergic cancer cells in the Dong et al. dataset.

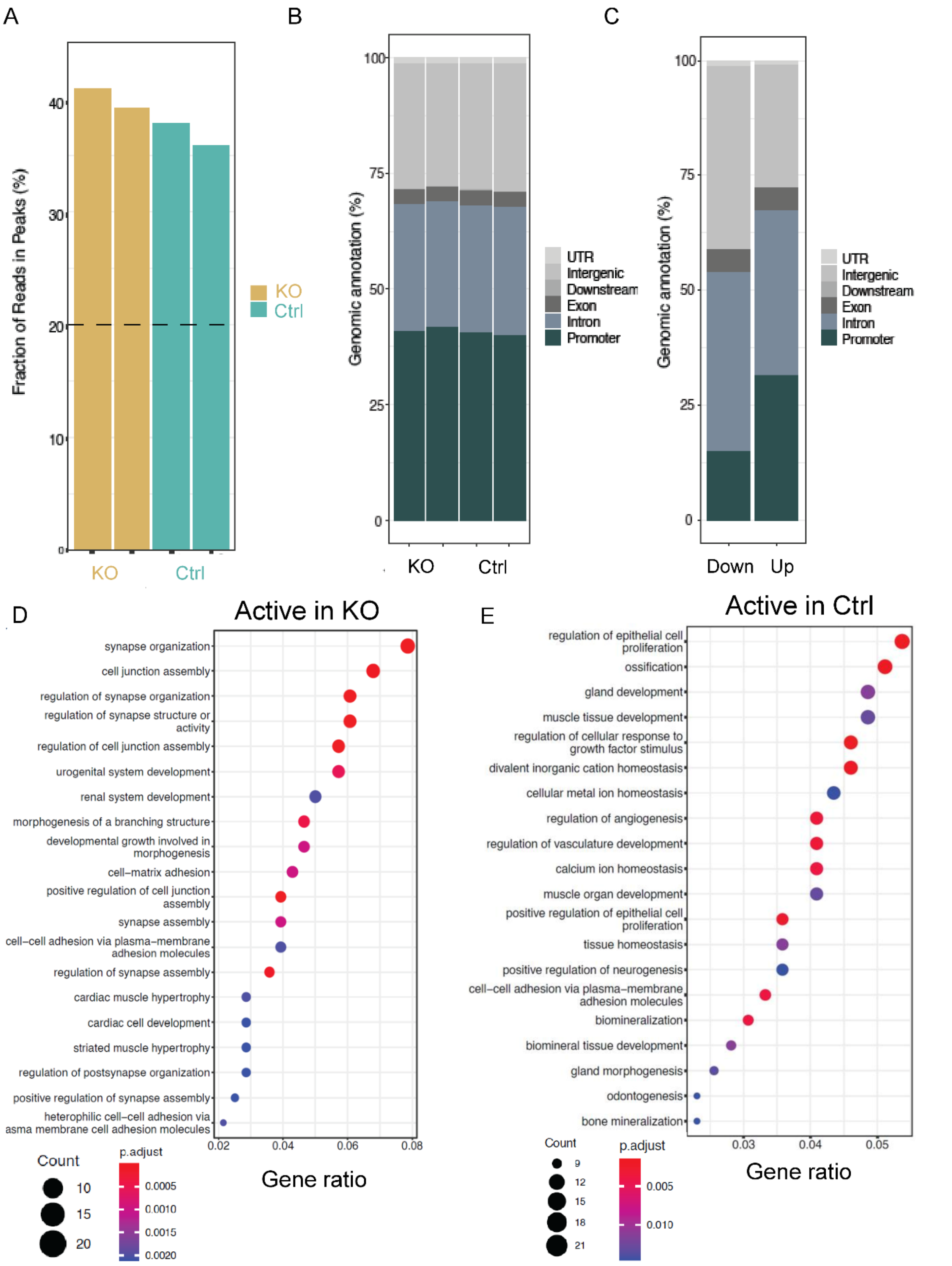

Figure S3. A) Fraction of reads in peaks (FRiP) in samples of *H2afy* CRISPR KO (KO) and control 9464D cells**.** Genomic annotation of **B)** all ATAC peaks or **C)** differential peaks in each mouse neuroblastoma sample. **D)** and **E)** Pathway enrichment analysis visualization of differential ATAC peaks in mouse neuroblastoma 9464D cells. The top 20 significantly enriched GO terms (P<0.05) in biological processes are displayed.

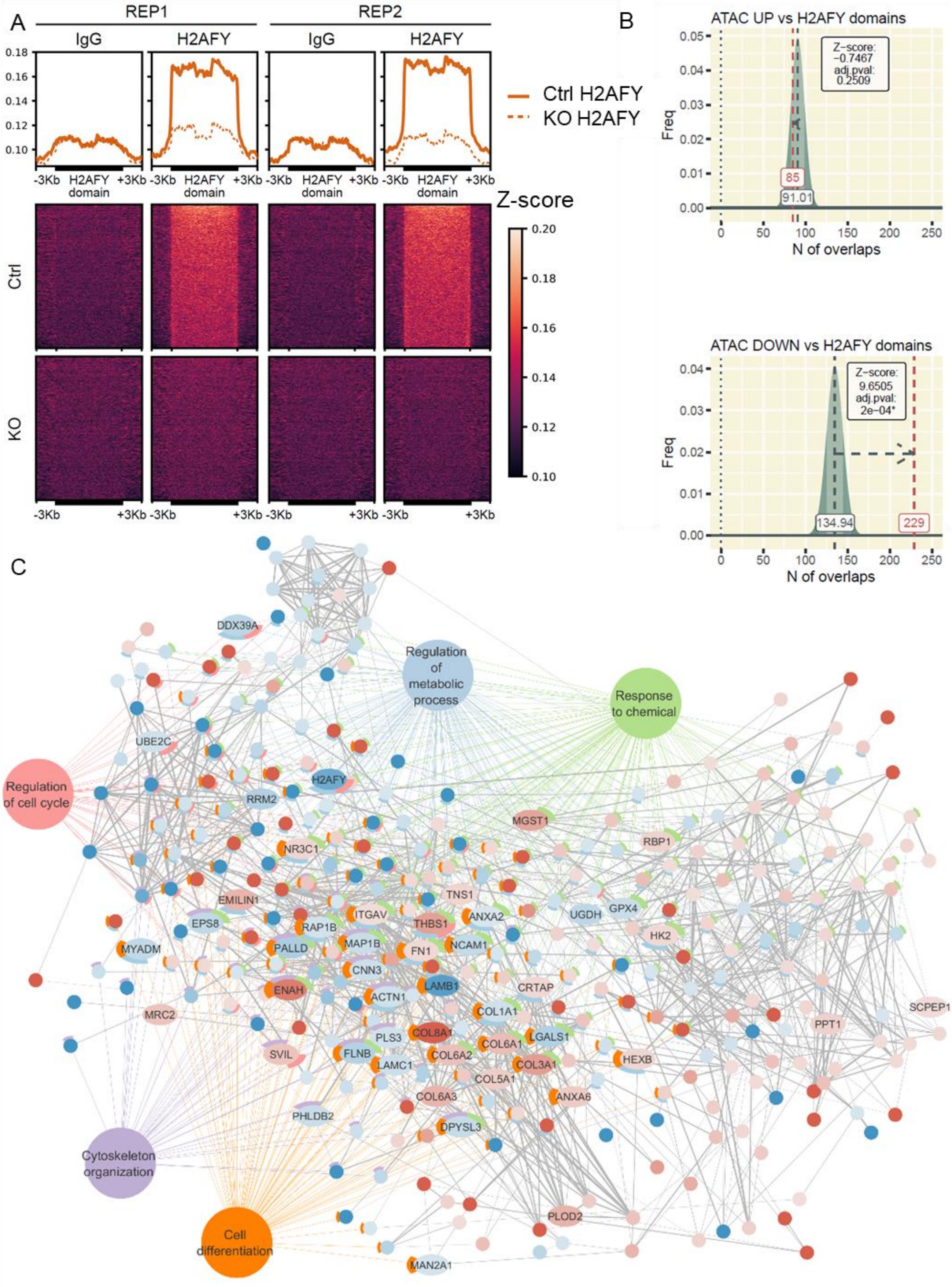
A
REP1
REP2
IgG
H2AFY
Ctrl H2AFY
KO H2AFY
-3Kb
H2AFY domain
+3Kb
Z-score
Ctrl
KO
B
ATAC UP vs H2AFY domains
Z-score: −0.7467 adj.pval: 0.2509
85
91.01
Freq
N of overlaps
ATAC DOWN vs H2AFY domains
Z-score: 9.6505 adj.pval: 2e-04*
134.94
229
C
Regulation of metabolic process
Response to chemical
Regulation of cell cycle
Cytoskeleton organization
Cell differentiation

Figure S4. A) Heatmap and mean profile visualization of the H2AFY CUT&RUN signal in 9464D cells across enriched domains, called with epic2 in two different experimental replicates. Domain calling was performed using the KO samples as a negative control. Every region was scaled to the same size and extended +/- 3kb in each side. A non-targeting IgG isotype antibody was used as a negative control. **B)** Permutation test results evaluating the genomic overlap between differential ATAC-Seq peaks in 9464D (UP or DOWN) and enriched domains of H2AFY. The green curve represented the random distribution of overlaps after randomizing the position of the ATAC-Seq peaks 5000 times. **C)** Network analysis of the label-free proteomics data in *H2afy* KO/ctrl 9464D cell line pair and the most relevant enriched pathways and different protein clusters were visualized. Color of the node represents the log2FC between KO and ctrl cells. Red represented upregulated proteins and blue represented downregulated proteins in KO cells. The additional color on the proteins corresponded to the pathways represented by large colored circles.

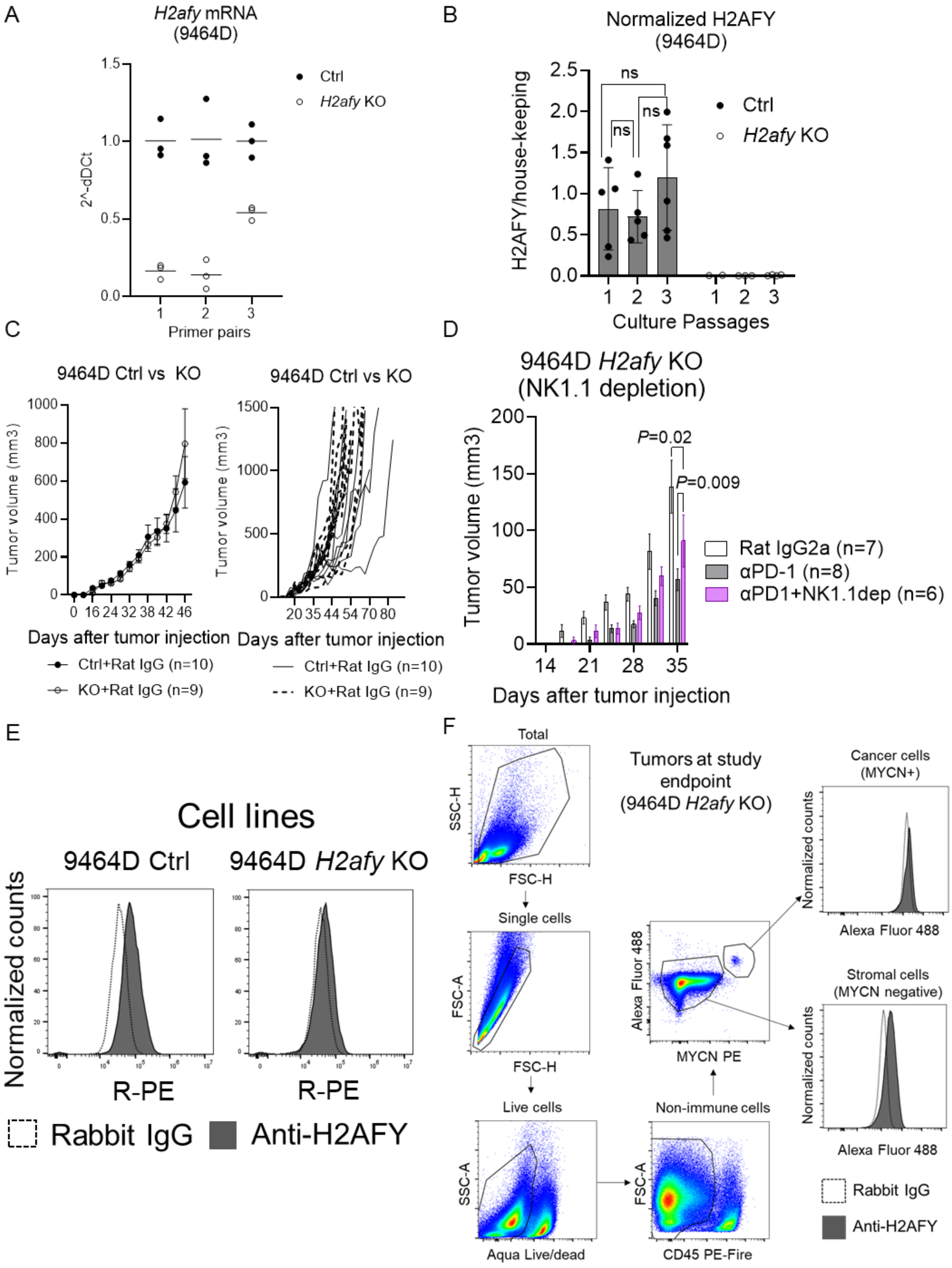
A
H2afy mRNA (9464D)
2^-dDCt
Primer pairs
Ctrl
H2afy KO
B
Normalized H2AFY (9464D)
H2AFY/house-keeping
ns
Ctrl
H2afy KO
Culture Passages
C
9464D Ctrl vs KO
Tumor volume (mm3)
Days after tumor injection
Ctrl+Rat IgG (n=10)
KO+Rat IgG (n=9)
D
9464D H2afy KO (NK1.1 depletion)
Tumor volume (mm3)
P=0.02
P=0.009
Rat IgG2a (n=7)
αPD-1 (n=8)
αPD1+NK1.1dep (n=6)
Days after tumor injection
E
Cell lines
9464D Ctrl
9464D H2afy KO
Normalized counts
R-PE
Rabbit IgG
Anti-H2AFY
F
Total
Single cells
Live cells
Non-immune cells
Tumors at study endpoint (9464D H2afy KO)
Cancer cells (MYCN+)
Stromal cells (MYCN negative)
Normalized counts
Alexa Fluor 488
MYCN PE
CD45 PE-Fire
Aqua Live/dead
Rabbit IgG
Anti-H2AFY

Figure S5. A) Expression of the *H2afy* mRNA was quantified in control or KO 9464D cells using qPCR. Each dot represented a technical replicate. **B)** Control or KO 9464D cells were harvested at different passages and expression of H2AFY was measured by western blotting. Protein expression was quantified by calculating the ratios between H2AFY and the house keeping proteins using the ImageJ software. At least 5 biological replicates were performed and each dot represents value of a biological replicate, ns: not significant, unpaired 2-tailed T-test. **C)** Average tumor volumes or individual tumor growth of *H2afy* KO or ctrl 9464D tumors in mice treated with the IgG control antibody. **D)** One day before IgG or αPD1 treatment, mice bearing *H2afy* KO 9464D tumors were treated with a depletion antibody against NK cells (clone PK136) at 100 μg per mouse (i.p.) every 6 days, 6-8 mice per group. Tumor growth was compared among groups using two-way ANOVA. **E)** The anti-H2AFY antibody or a rabbit isotype control IgG was conjugated with the Zenon R-PE dye and incubated with fixed/permeabilized control or *H2afy* KO 9464D cells. Fluorescence intensity was quantified using flow cytometry on live cells. Representative plots of 3 biological repeats were shown. **F)** *H2afy* KO 9464D tumors treated with the PD1 blockade was harvested at the study endpoint (>1000 mm^3) and the expression of H2AFY in MYCN+ NB cancer cells and stromal cells was quantified using flow cytometry. Gating strategy and representative plots of 3 mice were shown.

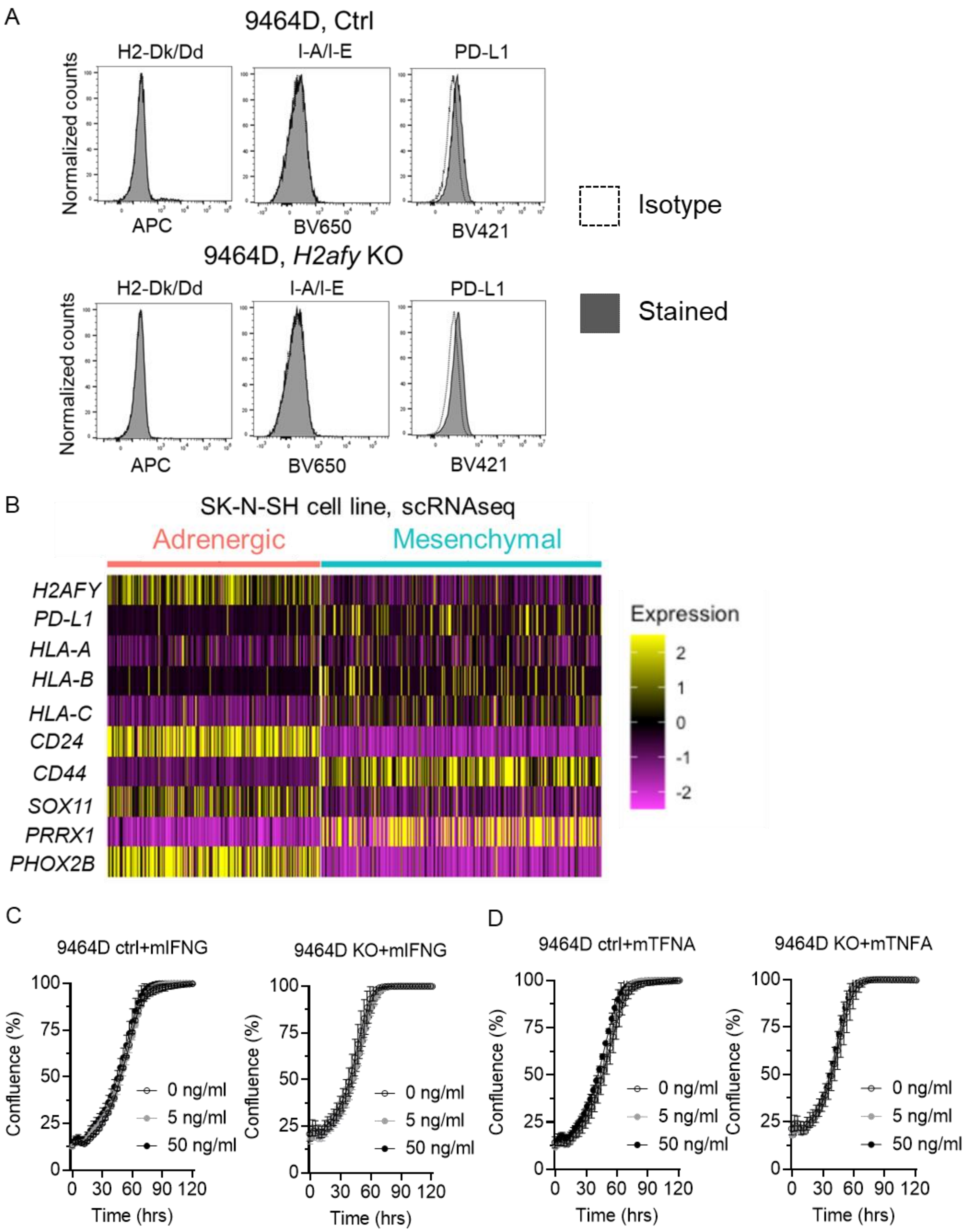

Figure S6. A) Control or H2afy KO 9464D cells were stained with fluorescence-conjugated antibodies against H2-Dk/Dd, I-A/I-E or PD-L1 and surface protein expression was quantified using flow cytometry. Corresponding isotype control antibodies were used in the same experiment. Representative plots of 3 biological repeats were shown. **B)** Expression of mRNAs associated with adrenergic or mesenchymal cell states, as well as immune-related genes was visualized in the SK-N-SH neuroblastoma cell line using a public scRNA-seq dataset (GSE229224). Control or *H2afy* KO 9464D cells were treated with **C)** recombinant murine IFNG (mIFNG) or **D)** mTNFA and cell proliferation was measured using live-cell imaging. Representative data of 2 biological repeats were shown.

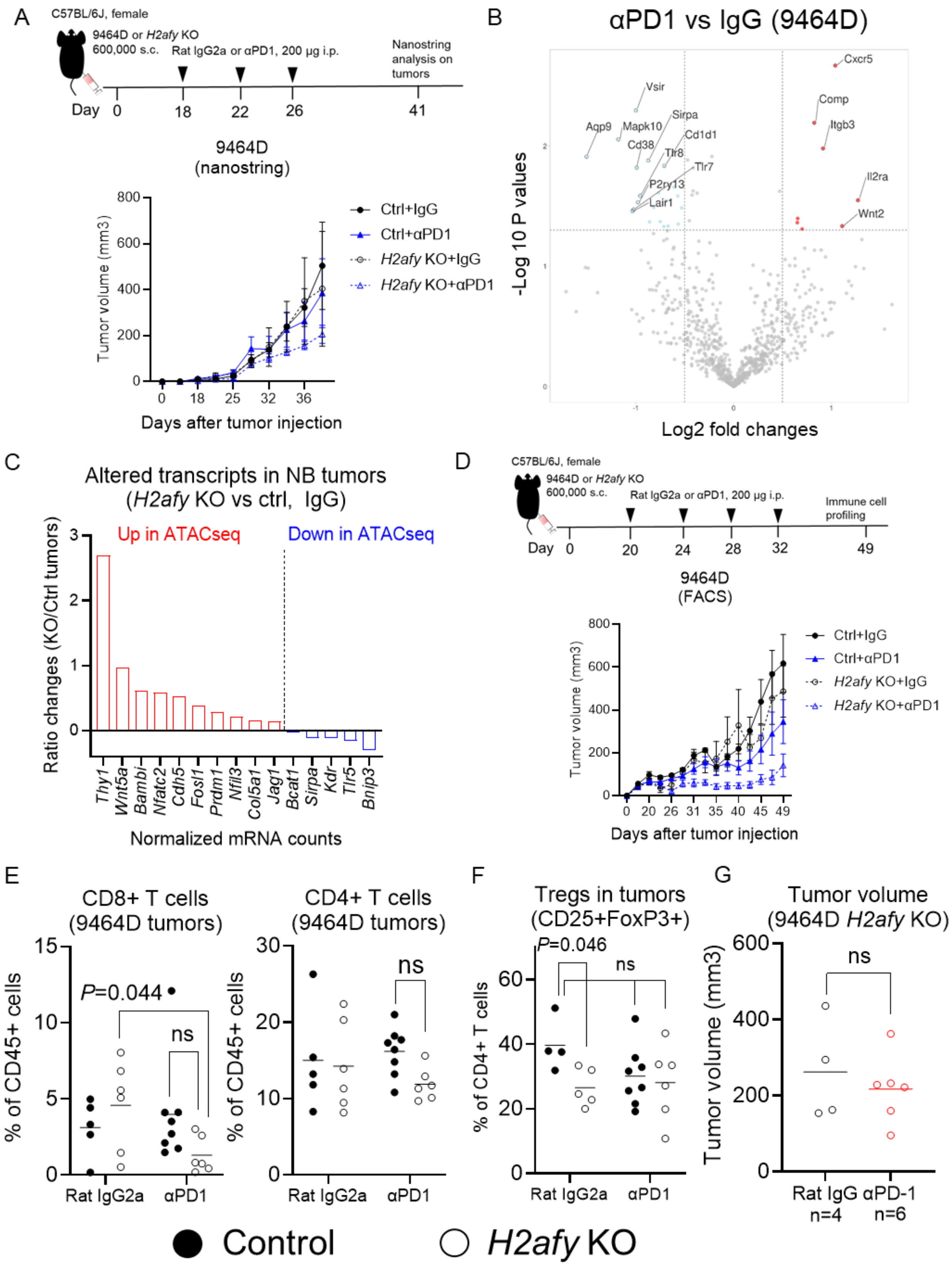

A
C57BL/6J, female
9464D or H2afy KO
600,000 s.c.
Rat IgG2a or αPD1, 200 μg i.p.
Nanostring analysis on tumors
Day 0 18 22 26 41
9464D (nanostring)
Tumor volume (mm3)
Days after tumor injection
Ctrl+IgG
Ctrl+αPD1
H2afy KO+IgG
H2afy KO+αPD1
B
αPD1 vs IgG (9464D)
-Log 10 P values
Log2 fold changes
Vsir
Aqp9
Mapk10
Sirpa
Cd38
Cd1d1
Tlr8
Tlr7
P2ry13
Lair1
Cxcr5
Comp
Itgb3
Il2ra
Wnt2
C
Altered transcripts in NB tumors (H2afy KO vs ctrl, IgG)
Up in ATACseq
Down in ATACseq
Ratio changes (KO/Ctrl tumors)
Thy1
Wnt5a
Bambi
Nfatc2
Cdh5
Fosl1
Prdm1
Nfil3
Col5a1
Jag1
Bcat1
Sirpa
Kdr
Tlr5
Bnip3
Normalized mRNA counts
D
C57BL/6J, female
9464D or H2afy KO
600,000 s.c.
Rat IgG2a or αPD1, 200 μg i.p.
Immune cell profiling
Day 0 20 24 28 32 49
9464D (FACS)
Tumor volume (mm3)
Days after tumor injection
Ctrl+IgG
Ctrl+αPD1
H2afy KO+IgG
H2afy KO+αPD1
E
CD8+ T cells (9464D tumors)
% of CD45+ cells
P=0.044
ns
CD4+ T cells (9464D tumors)
ns
Rat IgG2a
αPD1
F
Tregs in tumors (CD25+FoxP3+)
P=0.046
ns
% of CD4+ T cells
Rat IgG2a
αPD1
G
Tumor volume (9464D H2afy KO)
ns
Tumor volume (mm3)
Rat IgG n=4
αPD-1 n=6
Control
H2afy KO

Figure S7. A) Treatment schedule and average tumor volumes (mean±SEM) in mice bearing control or *H2afy* KO 9464D tumors for the nanostring analysis. **B)** Expression of mRNAs when comparing control 9464D tumors treated with the PD1 blockade or the isotype control. **C)** Up-regulated or down-regulated genes when comparing mRNA expression in KO and control 9464D tumors harvested from mice and ATAC-seq peaks on the cell line pair. **D)** Treatment schedule and average tumor volumes (mean±SEM) in mice bearing control or *H2afy* KO 9464D tumors for the flow cytometry analysis. **E)** Frequencies of tumor infiltrating CD8+ or CD4+ T cells or **F)** regulatory T cells (CD4+CD25+FoxP3+) in control or KO 9464D tumors receiving IgG or PD1 blockade. Each dot represents an individual mouse and statistical differences among groups were tested using 2-way ANOVA, ns: not significant. **G)** Comparison of tumor volumes of KO 9464D tumors treated with the IgG control (n=4) or PD1 blockade (n=7) at the point of flow cytometry analysis. Each dot represents an individual mouse, ns: not significant, unpaired 2-tailed T-test.

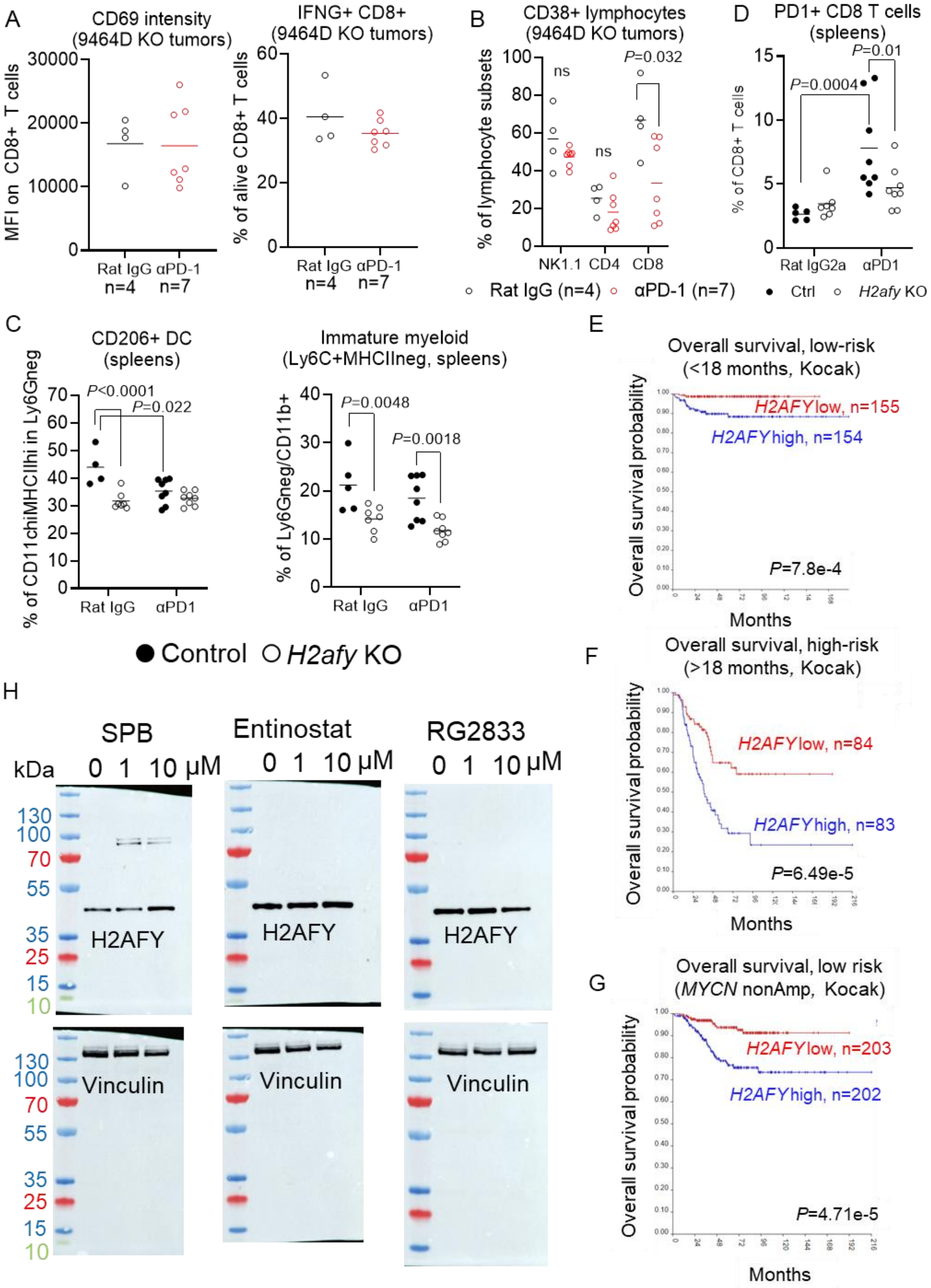

Figure S8. A) Expression intensity of CD69 and frequencies of IFNG+ were measured on tumor-infiltraing CD8+ T cells from size-matched *H2afy* KO 9464D tumors treated with IgG (n=4) or PD1 blockade (n=7). **B)** Frequencies of CD38+ cells in tumor-infiltrating CD8+ T cells, NK cells and CD4+ T cells from size-matched *H2afy* KO 9464D tumors treated with IgG (n=4) or PD1 blockade (n=7). Each dot represented an individual mouse and differences were tested using an unpaired 2-tail student T-test. Frequencies of **C)** CD206+ dendritic cells or immature myeloid cells, as well as **D)** PD1+ CD8+ T cells in spleens from control or KO 9464D

tumors receiving IgG or PD1 blockade, 4-8 tumors per group. Each dot represented an individual mouse and statistical differences among groups were tested using 2-way ANOVA. The prognostic value of *H2AFY* mRNA was tested in **E)** low-risk (<18 months) or **F)** high-risk (>18 months) NB patients or **G)** patients without *MYCN* amplification from the Kocak cohort. Median expression was used as a cut-off for the Kaplan-Meier curves. Survival difference was tested using the Log-rank test. **H)** 9646D cells treated for 48 hours with HDAC inhibitors at 10 or 1 μM in a 6-well plate with DMSO as control wereanalysed for expression of H2AFY by western blotting. A representative image of 2 biological repeats was shown.

Supplementary Tables

Supplementary Table 1: Antibodies

Name	Clone	Application	Product information
Anti-human/mouse H2AFY	Rabbit IgG	WB	Abcam/ab183041
Anti-human/mouse MYCN	Mouse IgG	WB	Santa Cruz/sc-53993
Anti-Rabbit IgG HRP-linked Antibody	Goat IgG	WB	Cell Signaling Technology/7074S
Anti-mouse IgG HRP-linked-Antibody	Horse IgG	WB	Cell Signaling Technology/7076S
Anti-human/mouse Vinculin	nVin-1, mouse IgG1	WB	Sigma Aldrich/V9131
Anti-human/mouse GAPDH	D6, IgG2b k light chain	WB	Santa Cruz/166545
PE-Cy7 anti-mouse CD206	C068C2, rat IgG2a, k	FACS	Biolegend/141720
Brilliant violet 650 anti-mouse I-A/I-E	M5/114.15.2, rat IgG2b, k	FACS	Biolegend/107641
PE-Dazzle 594 anti-mouse/human CD11b	M1/70, rat IgG2b, k	FACS	Biolegend/101256
APC-Cy7 anti-mouse Ly6G	1A8, rat IgG2a, k	FACS	Biolegend/127624
PerCp-Cy5.5 anti-mouse Ly6C	HK1.4, rat IgG2c, k	FACS	Biolegend/128012
PE anti-mouse CD11c	N418, Armenian hamster IgG	FACS	eBioscience/12-0114-83
Brilliant violet 421 anti-mouse PD-L1	10F.9G2, rat IgG2b, k	FACS	Biolegend/124315
Brilliant violet 605 anti-mouse CD86	GL-1, rat IgG2a, k	FACS	Biolegend/105037
FITC anti-mouse CD73	TY/11.8, rat IgG1, k	FACS	Biolegend/127219
PE anti-mouse CD4	RM4-5, rat IgG2a, k	FACS	Biolegend/100512
PE-Cy7 anti-mouse CD25	PC61, rat IgG1, λ	FACS	Biolegend/102016
APC-Cy7 anti-mouse CD38	90, rat IgG2a, k	FACS	Biolegend/102728
PerCP-Cy5.5 anti-mouse CD3	17A2, rat IgG2b, k	FACS	Biolegend/100218
PE anti-mouse TCF1	C63D9/rabbit IgG	FACS	Cell Signaling/14456S
AlexaFluor 700 anti-mouse CD172a (SIRPα)	P84, Rat IgG1, κ	FACS	Biolegend/144022
APC anti-mouse F4/80	BM8, Mouse IgG2a, κ	FACS	Biolegend/ 123116
APC anti-human HLA-ABC	W6/32, Mouse IgG2a, κ	FACS	Biolegend/311410
FITC anti-human CD274	MIH2, Mouse IgG1, κ	FACS	Biolegend/393605
APC/Cyanine7 anti-human HLA-DR	L243, Mouse IgG2a, κ	FACS	Biolegend/307617
PE anti-human CD119 (IFN-γ R α chain)	GIR-208, Mouse IgG2a, κ	FACS	Biolegend/308606
Anti-mouse CD16/32	93, Rat IgG2a, λ	FACS	Invitrogen/14-0161-81
PE-Fire700 anti-mouse CD45	30-F11, Rat IgG2b, κ	FACS	Biolegend/103178
Brilliant Violet 650 anti-mouse CD8a	53-6.7, Rat IgG2a, κ	FACS	Biolegend/100742
PE anti-mouse TNFa	MP6-XT22, Rat IgG1, κ	FACS	Invitrogen/12-7321-81
Brilliant Violet 421 anti-mouse CD279 (PD-1)	29F.1A12, Rat IgG2a, κ	FACS	Biolegend/135221
FITC anti-mouse CD3	17A2, Rat IgG2b, κ	FACS	Biolegend/100203
APC anti-mouse/rat FOXP3	FJK-16s, Rat IgG2a, κ	FACS	Invitrogen/17-5773-82
Spark UV 387 anti-mouse CD4	GK1.5, Rat IgG2b, κ	FACS	Biolegend/100492

PE-Cy5 anti-mouse CD69	H1.2F3, Armenian Hamster IgG	FACS	Invitrogen/15-0691-82
FITC anti-mouse H2Kd/Dd	34-1-2S, Mouse IgG2a, κ	FACS	Invitrogen/11-5998-81
Brilliant Ultra Violet 805 anti-mouse IFNG	XMG1.2, Rat IgG1, κ	FACS	Invitrogen/368-7311-82
PE anti-mouse MYCN	D4F9Z, Rabbit IgG	FACS	Cell Signaling/17150S
APC anti-mouse H2Kd/Dd	34-13-2S, Mouse IgG2a, κ	FACS	Affymetrix eBioscience /17-5998-82
Anti-mouse PD-1	RMP1-14, rat IgG2a, k	In vivo block	BioXcell/BE0146
Rat IgG2a isotype	2A3, rat IgG2a k	In vivo block	BioXcell/BE0089
Anti-mouse CD4	GK1.5, rat IgG2b, k	In vivo block	BioXcell/BE0003-1
Anti-mouse CD8α	2.43, rat IgG2b, k	In vivo block	BioXcell/BE0061
Anti-mouse NK1.1	PK136, mouse IgG2a, k	In vivo block	BioXcell/BE0036
macroH2A1 antibodies		CUT&RUN	Home-made/Buschbeck et al.
Rabbit Anti-Mouse IgG H&L	Rabbit IgG	CUT&RUN	Abcam/ab46540

Supplementary Table 2: other reagents

Name	Application	Product information
Lymphoprep	Cell isolation	StemCell/07851-07861
SepMate tubes	Cell isolation	StemCell/85450
CD14+ positive selection kit	Cell isolation	StemCell/17858
Red blood cell lysis buffer	Cell isolation	Biolegend/420301
Alt-R® S.p. Cas9 Nuclease V3	CRISPR KO	IDT/1081058
TracrRNA	CRISPR KO	IDT/1072534
ID TE buffer	CRISPR KO	IDT/11-01-02-02
Nuclease free duplex buffer	CRISPR KO	IDT/11-01-03-01
Entinostat (MS-275)	Molecular inhibition	Selleck Chemicals/S1053-10MG
RG2833	Molecular inhibition	Medchem Express/HY-16425
4-PBA (Sodium Phenylbutyrate)	Molecular inhibition	Selleck Chemicals/S4125-50MG
Aqua fixable live/dead marker	FACS	Invitrogen by Thermo Fisher Scientific/L34966A
CellTrace violet	FACS	Invitrogen by Thermo Fisher Scientific/C34557
FoxP3 staining buffer set	FACS	eBioscience/00-5523-00
True-Nuclear Fixation/Permeabilization Kit	FACS	BioLegend/424401
Zenon anti-Rabbit AF488 Dye	FACS	Invitrogen/Z25302 A
Zenon anti-Rabbit R-PE Dye	FACS	Invitrogen/Z25355 A
Rabbit IgG isotype	FACS	PeProTech/500-P00-500UG
Tumor dissociation kit, mouse	Tissue digest	Miltenyi Biotech/130-096-730
GentleMacs C-tubes	Tissue digest	Miltenyi Biotech/130-093-237
MACS smartstrainers	Tissue digest	Miltenyi Biotech/130-110-916
mIFNg	Cell culture	PeProTech/315-05-100UG
mTNFα	Cell culture	Gibco/PMC3014
rhIFNg	Cell culture	Peprotech/300-02
IMDM medium	Cell culture	Gibco/12440-053
Heat inactivated FBS	Cell culture	Gibco/10500-064
Penstrep	Cell culture	Gibco/15070063
MycoAlert® Mycoplasma Detection Kit	Cell culture	Lonza/LT07-318
WB gels (NuPAGE 4-12% Bis-Tris)	WB	Invitrogen by Thermo Fisher Scientific/NP0321BOX
Protein Ladder	WB	Thermo Scientific/26619
iBlot 2NC Regular Stacks	WB	Invitrogen by Thermo Fisher Scientific/IB23001
MOPS SDS Running Buffer (20x)	WB	Novex by life technologies/NP0001
Transfer Buffer (20x)	WB	Novex by life technologies/NP0006-1
LDL Sample buffer (4x)	WB	Novex by life technologies/B0007
SKIM Milk POWDER	WB	OXOID/LP0033
Ponceaus S solution (0.2%)	WB	Serva/33427.01
Pico substrate	WB	Thermo Scientific/34580
RIPA Buffer	WB	Thermo Scientific/89900
BCA Protein Assay Kit	WB	Thermo Scientific/23225
Protease Inhibitor Cocktail	WB	Thermo Scientific/78446
EveryBlot Blocking Buffer	WB	BioRad/12010020
MinElute PCR Purification kit	PCR	Qiagen/28004
AllPrep RNA/protein extraction kit	Nanostring	Qiagen/80004
RNAeasy Mini Kit	PCR	Qiagen/74104
SsoAdvanced Universal SYBR Green Supermix	PCR	Bio-Rad/1725271
iScript™ cDNA Synthesis Kit	PCR	Bio-Rad/ 1708890
Human IFNg ELISA HRP kit	ELISA	Mabtech/3420-1H-20
Human GranzymeB ELISA HRP kit	ELISA	Mabtech/ 3486-1H-20
Lysis buffer for ATAC sequencing	ATAC-seq	Invitrogen/15567-027

NaCl	ATAC-seq	Invitrogen/AM9759
MaCl2	ATAC-seq	Invitrogen/AM9530G
0.1% IGEPAL CA- 630	ATAC-seq	Sigma-Aldrich/13021-50
Tris-HCl	ATAC-seq	Invitrogen/15568-025
Nuclease-free water	ATAC-seq	Invitrogen/AM9932
Qiagen MinElute PCR Purification kit	ATAC-seq	Qiagen/28004
NEBNext High-Fidelity 2× PCR master mix	ATAC-seq	New England Biolabs/M0541S
SPRI beads	ATAC-seq	Beckman Coulter/B23317
CUTANA™ Concanavalin A-Conjugated Paramagnetic Beads	CUT&RUN	Epicypher/21-1401
Antibody binding buffer	CUT&RUN	Meers et al., 2019
CUTANA™ pA/G-MNase	CUT&RUN	Epicypher/15-1016
CaCl2	CUT&RUN	Merck/1023820500
STOP buffer containing chelating agents	CUT&RUN	Meers et al., 2019
Proteinase K	CUT&RUN	Merck/P6556-5MG
ChIP DNA Clean & Concentrator	CUT&RUN	Zymo Research/D5205
KAPA HyperPrep kit	CUT&RUN	Roche/ 07962363001
NEXTflex DNA barcodes for Illumina	CUT&RUN	Bioo Scientific/514102
KAPA Library Quantification kit	CUT&RUN	Roche/07960298001
NovaSeq X Plus instrument	CUT&RUN	Illumina

Supplementary Table 3: Sequences

**Name**	**Application**	**Sequence**
Mouse *H2afy* crRNA	CRISPR	GGCCAGCGCAGACAGTACGA
Carrier DNA	CRISPR	CCAGCAGAACACCCCCATCGGCGACGGCCCCGT GCTGCTGCCCGACAACCACTACCTGAGCACCCA GTCCGCCCTGAGCAAAGACCCCAACGAGA
Mouse *H2afy* Forward Primer 1	qPCR	TACAGACGGCTTCACTGTCC
Mouse *H2afy* Reverse Primer1	qPCR	GGTCAATGTCAGCATTGGTAGG
Mouse *H2afy* Forward Primer 2	qPCR	GCTTTGAGGTGGAGGCCATAA
Mouse *H2afy* Reverse Primer2	qPCR	GGATCACAAACTTGGCAGGC
Mouse *H2afy* Forward Primer 3	qPCR	CCTACCAATGCTGACATTGACC
Mouse *H2afy* Reverse Primer3	qPCR	TGCACCCCAGACAGGACTAT
Mouse *β-actin* Forward Primer	qPCR	ATGACGATATCGCTGCGCTGGT
Mouse *β-actin* Reverse Primer	qPCR	CCTCGTCACCCACATAGGAGTC

Supplementary methods

Cas9 transfection of IMR32 cells

IMR32 cells were lentivirally transduced with the pLenti-Cas9-T2A-Blast-BFP vector with 2 µg/ml polybrene to express codon-/optimised WT Sp Cas9 linked to blasticidin-S-deaminase-mTagBFP fusion protein (derived from lenti-dCAS9-VP64_Blast, a gift from Feng Zhang, Broad Institute of MIT and Harvard, Boston, USA, Addgene #61425). Following blasticidin selection, stable BFP-expressing cells were repeatedly sorted by fluorescence-activated cell sorting (FACS).

Guide RNA library transduction

The Brunello sgRNA library (1) was synthesised as 79 bp long oligos (indicated in bold in the sequence below, CustomArray, Genscript). The pool of oligos was generated as double-strands by PCR to include an A-U flip in the tracrRNA (2), 10-nucleotide long random Unique Molecular Identifiers, and an i7 sequencing primer binding site(3).

ggctttatatat**cttgtggaaaggacgaaacaccgnnnnnnnnnnnnnnnnnnnngtttaagagctagaaatagcaagtttaaataaggct**agtccgttatcaacttgaaaaagtggcaccgagtcggtgcttttttGATCGGAAGAGCACACGTCTGAACTCCAGTCACNNNNNNNNNNaagcttggcgtaactagatcttgagacaaa

The PCR product carrying the sequence was cloned into pLenti-Puro-AU-flip-3xBsmBI by Gibson assembly [3], and confirmed by DNA sequencing before packaging into lentivirus. The functional viral titer was determined by the fraction of puromycin-resistant cells after transduction with varying amounts of the lentiviral library. Cas9-expressing IMR32 cells were then transduced in duplicate with the Brunello gRNA library at an approximate multiplicity of infection (MOI) of 0.3-0.4 with a coverage of 1,000 cells per guide in the presence of 2 µg/ml of polybrene. To ensure sufficient coverage, a minimum of 80 million cells were needed. Transduced cells were selected by culturing from day 2 to 10 in the presence of 2 µg/ml puromycin.

Whole-genome CRISPR screens in TICS

Library-transduced Cas9^{+} IMR32 cells were cultured overnight in T175 flasks with IMDM complete media prior to setting up of co-culture systems. The following day, CD14-depleted fresh human lymphocytes were added to library-transduced Cas9^{+} IMR32 cells, with or without nivolumab at 10 µg/ml. HLA typing was not possible in order to maintain the anonymity of the blood donors. Cas9-expressing IMR-32 cells cultured alone were used as controls. On day 5, floating cells were gently washed off and adherent cancer cells were harvested for genomic DNA isolation using QIAmp DNA Blood Maxi kit (Qiagen) for next-generation sequencing. Guide cassettes were amplified by PCR using modified primers as described in Schmierer et al 2017(3),

PCR2_fw acactctttccctacacgacgctcttccgatctcttgtggaaaggacgaaacac and

PCR3_fw aatgatacggcgaccaccgagatctacac [i5] acactctttccctacacgacgctct.

The resulted amplicons were sequenced using Illumina NovaSeq with a Read 1 of 20 cycles using custom primer *CGATCTCTTGTGGAAAGGACGAAACACCG* supported by dual indexing of 10 cycles and 6 cycles to read i7 Unique Molecular Identifiers (UMIs) and i5sample barcode respectively.

Data analysis of CRISPR screens

NGS data was analyzed using MaGeCK software (4) and by UMI lineage dropout analysis (3). A list of mitochondrial and ribosomal genes (n=638) was obtained using the R package biomaRt (MT, rRNA, rRNA_pseudogene, and ribozyme biotypes) and excluded to reduce total gene search space. For each comparison, gene essentiality scores were determined for individual gRNA using MAGeCK. Depleted genes were selected based on the distribution of essentiality scores using a predefined cut-off, i.e. mean-2 standard deviation (SD) for donor 1 (-0.22) and donor 2 (-0.24), respectively.

Depleted genes were tested against GO (biological process) database (5, 6) to identify over-represented pathways. The EnrichAnalyzer function from the MAGeCKFlute R package was used to perform the hypergeometric test with Benjamini-Hochberg adjusted p-value cut-off of 0.25.

Deletion of the *H2afy* gene using CRISPR/Cas9

Murine NB cell line 9464D was transfected with ribonucleoprotein (RNP) complexes containing gene-specific crRNA, tracrRNA, and a recombinant Cas9 protein (all from IDT) using an optimized electroporation program. For every transfection, guide RNA (gRNA) was formed first by annealing 1 µl of *H2afy*-specific crRNA (100 µM) with 1 µl of tracrRNA (100 µM) in 1.7 µl IDT duplex buffer by boiling at 95°C for 5 minutes and cooled down to 4°C. Then RNP was formed by incubating 1 µl of Cas9 protein (10 mg/ml) to the gRNA at room temperature for 15-20 minutes. Moreover, 0.3 µl of carrier DNA (100 µM, IDT) was added to improve the efficacy of gene editing. RNP complexes without the crRNAs were transfected into cells as controls. Next, freshly harvested NB cells ($4x10^{5}$) were re-suspended in 5 µl of buffer R and mixed with 5 µl of RNP solution before electroporation. Transfected NB cells were rested for at least 2-3 days before assessing gene expression levels. Repeated introduction of the RNP complex was performed to increase protein deletion in the cell line pool.

Proteomics and data analysis

9464D control and *H2afy* KO cells lysed using a lysis buffer containing 100 µl of 1% β-octyl glucopyranoside and 6M urea followed by sonication with 1 pulse at 40% amplitude using a 3 mm probe for 30 seconds. After homogenization, samples were incubated for 1 hour at 4°C with gentle agitation. Then, samples were centrifuged at 14,000 rpm for 10 min and precipitates were pressed to collect more supernatant containing extracted proteins. Protein concentrations were determined using DC protein assay with BSA standards and 35 µg of protein was taken for digestion. Proteins were further reduced, alkylated and digested with trypsin on-filter using 3kDa spin filter (Millipore) and air-dried using SpeedVac system. Samples were then dissolved in 100 µl of 0.1% formic acid and diluted 4x prior to LC-MS/MS analysis. C18 columns were used to separate peptides in a reverse phase with 150-min gradient and electro-sprayed onto Q-Exactive Plus Mass Spectrometry (Thermo Finnigan). Tandem mass spectrometry was performed using higher energy collision dissociation and the raw data were analysed and annotated using MaxQuant software.

Quadruplicates from each 9464D control and KO sample group were analyzed by comparing the LFQ intensities. Proteins detected in at least 3 replicates in one group but not found in the other were classified as unique. Additionally, proteins identified in at least 2 replicates within a group were included for differential expression analysis. To quantify the difference in protein abundance between sample groups, average LFQ intensities were calculated within each groups, and then converted to log2 fold change. Statistical significance of the protein change were determined using a two-sample T-test, with Welch correction applied to account for uneven sample sizes when the protein was not identified in all the four samples per group. The p values were adjusted using the False Discovery Rate (FDR). Threshold for differential protein expression was set at an absolute log2FC above 0.5 and an FDR below 0.05. To determine the enriched pathways, overrepresentation analysis using clusterProfiler was carried out for downregulated and upregulated proteins separately, querying the Hallmarks, Reactome and Gene Ontology Biological Processes databases (p adjusted < 0.05). Unique and differentially expressed proteins were further investigated using STRING (https://string-db.org), focusing on proteins with at least one interacting neighbor, visualized using Cytoscape (v3.10.0). STRING enrichment was employed to assess network pathway enrichment (FDR < 0.05).

References

1. Doench JG, Fusi N, Sullender M, Hegde M, Vaimberg EW, Donovan KF, et al. Optimized sgRNA design to maximize activity and minimize off-target effects of CRISPR-Cas9. *Nat Biotechnol.* 2016;34(2):184-91.
2. Cross BC, Lawo S, Archer CR, Hunt JR, Yarker JL, Riccombeni A, et al. Increasing the performance of pooled CRISPR-Cas9 drop-out screening. *Sci Rep.* 2016;6:31782.
3. Schmierer B, Botla SK, Zhang J, Turunen M, Kivioja T, and Taipale J. CRISPR/Cas9 screening using unique molecular identifiers. *Mol Syst Biol.* 2017;13(10):945.
4. Li W, Xu H, Xiao T, Cong L, Love MI, Zhang F, et al. MAGeCK enables robust identification of essential genes from genome-scale CRISPR/Cas9 knockout screens. *Genome Biol.* 2014;15(12):554.
5. Ashburner M, Ball CA, Blake JA, Botstein D, Butler H, Cherry JM, et al. Gene ontology: tool for the unification of biology. The Gene Ontology Consortium. *Nat Genet.* 2000;25(1):25-9.
6. Gene Ontology C, Aleksander SA, Balhoff J, Carbon S, Cherry JM, Drabkin HJ, et al. The Gene Ontology knowledgebase in 2023. *Genetics.* 2023;224(1).
