# Supplementary figures and images for: Epigenetic regulation of cell state by H2AFY governs immunogenicity in high-risk neuroblastoma
Source: J Clin Invest. 2024 Sep 10;134(21):e175310. doi: 10.1172/JCI175310 (PMC11527455; doi:10.1172/JCI175310)

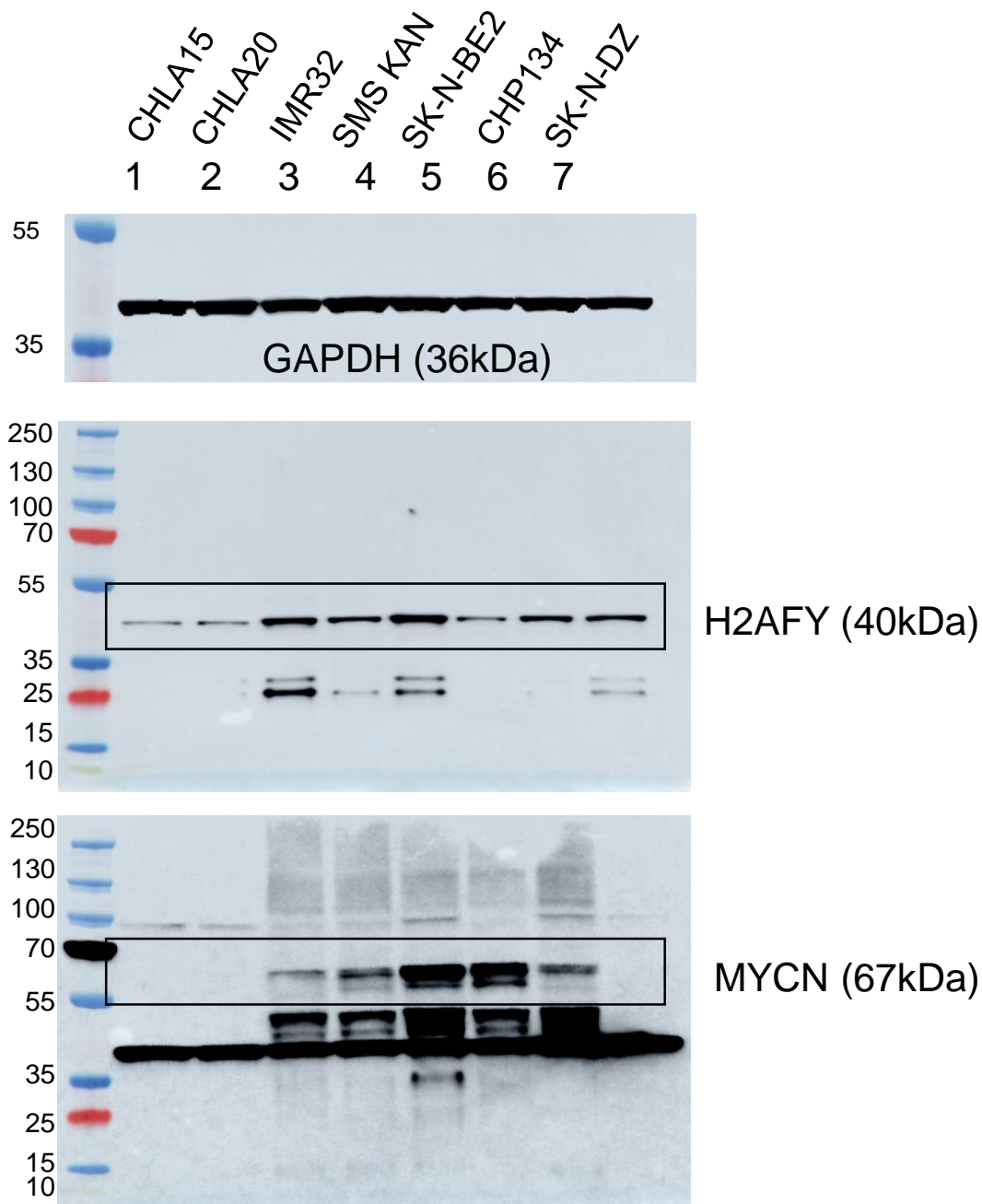

Uncut membrane for Figure 1A and 2G

# 9464D

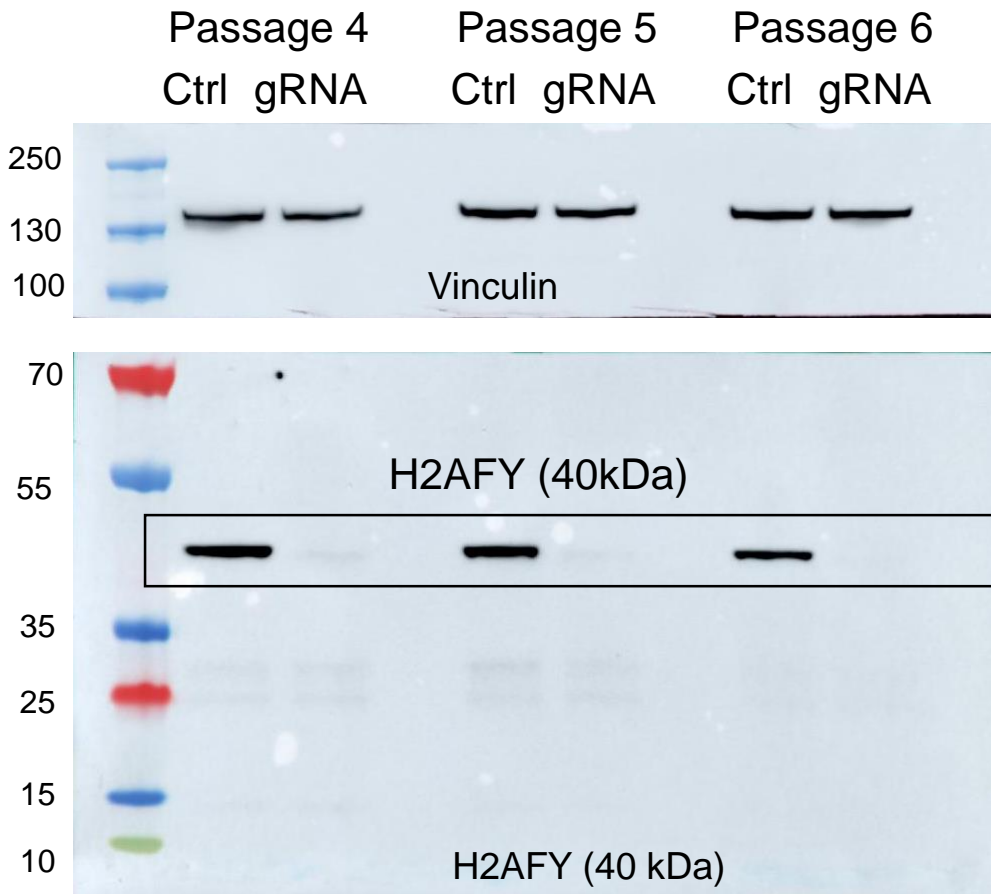

Uncut membrane for Figure 5A

Supplement: Unedited blot and gel images [file jci-134-175310-s047.pdf]
